# Trends in mental health care in Brazilian Primary Health Care before, during, and after COVID-19: an ecological interrupted time-series study (2018-2023)

**DOI:** 10.1590/1980-549720260037

**Published:** 2026-07-31

**Authors:** Lucas Oltramari, Milenne Souza de Lima, Eduardo Viegas da Silva

**Affiliations:** I Escola de Saúde Pública do Rio Grande do Sul - Porto Alegre (RS), Brazil.; II Secretaria Municipal de Saúde de Cachoeirinha - Cachoeirinha (RS), Brazil.; III Secretaria Estadual de Saúde do Rio Grande do Sul - Porto Alegre (RS), Brazil.

**Keywords:** Mental disorders, Primary health care, Health services research, COVID-19, Interrupted time series analysis

## Abstract

**Objective:**

To evaluate the temporal evolution of mental disorder-related visits in Primary Health Care (PHC) within the Brazilian Unified Health System (SUS) and estimate the effects of social distancing, the Covid-19 pandemic, and the post-pandemic period.

**Methods:**

This is a national ecological interrupted time-series study using data from the Primary Health Care Information System (SISAB/DATASUS). Monthly proportional and population incidence rates of mental disorder-related visits (ICD-10 F00-F99) in PHC were analyzed. Segmented regression models with negative binomial distribution and autoregressive structure were fitted.

**Results:**

A total of 38.7 million PHC visits were analyzed. In the pre-pandemic period, the share of mental disorder-related visits increased (exp(β)_Trend_=1.011; 95%CI 1.008-1.014). During social distancing, the proportion of these visits increased abruptly (exp(β)_Level_=1.241; 95%CI 1.149-1.339), largely associated with a reduction in the total volume of PHC visits. During the rest of the pandemic, the proportional increase plateaued. We observed regional heterogeneity (p<0.001), with greater level increase in the Northeast. In the post-pandemic period, growth resumed in both the proportion and the population incidence of mental disorder-related visits; increases were notable for anxiety, neurodevelopmental and child and adolescent disorders.

**Conclusion:**

The share of mental disorder-related visits in Brazilian PHC increased over the study period. Social distancing resulted in an abrupt proportional increase in the context of reduced overall visits, followed by heterogeneous recomposition across regions and diagnostic groups. These findings indicate a recent increase in demand for mental health care in PHC.

## INTRODUCTION

Among Primary Health Care (PHC) users, there is a high prevalence of mental disorders^1^ ^,^ ^2^ ^,^ ^3^, especially common mental disorders (CMD) such as anxiety and depression. Mental disorders are also among the main responsible for the global burden of disease, especially in Brazil, which leads indicators of years lived with disability due to anxiety disorders and bipolar disorder in the Americas^4^ ^,^ ^5^ ^,^ ^6^.

In the context of the Brazilian Unified Health System (SUS), mental health care is organized by the Psychosocial Care Network (*Rede de Atenção Psicossocial* - RAPS), which integrates multiple care centers. While the Psychosocial Care Centers (*Centros de Atenção Psicossocial* - CAPS) manage severe and persistent cases, PHC is responsible for the care of CMD and the longitudinal monitoring of stabilized cases, playing a strategic role in early detection and coordination of care-in line with the recommendations of the World Health Organization (WHO) to foster community services, especially in middle-income countries^7^ ^,^ ^8^.

Given the central role of PHC in the coordination of care for mental disorders within the RAPS, it is worth understanding how these disorders are manifested in the care production of this level of care. Although there are several qualitative studies on mental health care in PHC^9^, the national literature is still scarce regarding the frequency and proportional share of these services over time.

Moreover, the Covid-19 pandemic represented a disruption in health systems, including periods of social distancing and reorganization of supply, with relevant impacts on the mental health of the population^10^ and the use of services^11^ ^,^ ^12^. According to international studies, there have been specific increases in the proportional demand for mental disorder-related visits in PHC during the first months of the pandemic^13^. In Brazil, there is evidence of increasing inequalities in mental health symptoms during the pandemic. The period of social distancing caused financial losses, especially among families with lower socioeconomic status. This and other exposures-related to the spread of the SARS-CoV-2 virus and the measures implemented to contain abrupt increases in the incidence of Covid-19-possibly influenced the prevalence of CMD at the population level, which potentially increased the need for care in PHC^14^. However, it remains uncertain how this context was reflected in the organization and profile of the services provided in PHC of the SUS, particularly with regard to the relative share of mental disorders in the provided care.

Thus, in the present study, we aimed at evaluating the temporal evolution of the proportional share rate and the population incidence rate of mental disorder-related visits in PHC, for Brazil and its macroregions, estimating the effects of the most critical period of social distancing, the Covid-19 pandemic, and the post-pandemic period. In addition, by stratifying groups of mental disorders, we sought to identify possible differences between their specific temporal patterns.

## METHODS

### Study design and data sources

This is an ecological, interrupted time-series study^15^, with population data on mental disorder-related visits in PHC of the SUS, monthly, between January 2018 and December 2023. The study has national coverage, comprising Brazil and its five macroregions. Information was extracted from the “Individual Production” section of the Primary Health Care Information System (*Sistema de Informações em Saúde para a Atenção Básica* - SISAB) of the SUS Department of Informatics (*Departamento de Informática do SUS* - DATASUS), which consolidates records of care provided directly by PHC professionals in the e-SUS APS (a strategy from the Brazilian Ministry of Health to computerize and restructure information on PHC).

### Data collection instruments

For data collection, an automated scraping strategy was used, programmed in Python (version 3.12), to ensure comprehensiveness and reproducibility in access to the available information. Data were extracted in May 2025.

### Variables and definitions

For all analyses, the data were stratified into:

1. Geographic unit: mental disorder-related visits in Brazil and in each macroregion; and
2. Mental and behavioral disorder-related visits, aggregated at group level according to ICD-10, at national level.

The study was segmented into four phases:

1. Pre-pandemic (Jan./2018-Mar./2020);
2. Social distancing (Apr.-June/2020);
3. Pandemic (July/2020-Mar./2022); and
4. Post-pandemic (Apr./2022-Dec./2023).

The periods were defined based on the identification of sustained structural rupture as of April 2020 and the main national regulatory frameworks (Legislative Decree No. 6/2020; GM/MS [Brazilian Ministry of Health Office] Ordinance No. 356/2020; GM/MS Ordinance No. 913/2022). Social distancing was analyzed separately due to the sharp and transient drop in the total volume of visits, potentially capable of distorting the estimates of the subsequent pandemic phase.

The outcome variable was the monthly proportional rate of records with main diagnosis of mental disorders (CID-10 F00-F99), calculated as the number of mental disorder-related visits divided by the total number of general visits and multiplied by one thousand. The indicator allows us to evaluate the relative share of these disorders in the set of care services provided in PHC over time. The independent variables included continuous time in months since January 2018, as well as indicators that captured changes in level and trend associated with segmentations by period phase.

### Statistical analysis

#### Descriptive analysis

The descriptive analysis consisted of the presentation of absolute and relative measures of mental disorder-related visits in PHC. Medians and interquartile ranges (IQR) were arranged for temporal segments. The proportions and proportional rates of visits were calculated in relation to the total visits in PHC in the respective geographic unit.

#### Segmented regression model for proportional rates of visits

To quantify the impact of social distancing, the pandemic, and the post-pandemic period on the proportional rates of mental disorder-related visits in PHC, mixed generalized linear models were adjusted with quasi-likelihood estimation, using the *glmmPQL* function of the *nlme* package, specified with negative binomial distribution and logarithmic link, considering the observed overdispersion. The dispersion parameter was estimated iteratively based on the method of moments. The model included an ARMA(1,1) correlation structure in residuals over time. To control seasonal patterns, *Fourier* harmonic terms were included in the fixed component of the model. Each model used as a dependent variable the monthly number of mental disorder-related visits, including as offset the logarithm of the total number of general visits in the same period.

To formally evaluate regional differences in response to the segmented periods, a complementary model was adjusted by *glmmTMB,* with negative binomial distribution and AR(1) correlation structure in the residuals, including the “region” variable as fixed effect and terms of interaction between region and temporal phase indicators. The comparison between models with and without interaction was performed by the likelihood ratio test (5% significance level). All analyses were performed in *R* (v.4.5.0).

The level change coefficients were interpreted as structural changes in proportion, while trend change reflected gradual changes, both compared to the pre-pandemic period. For the social distancing period, only an immediate change in level was modeled, without estimate of trend, due to its short duration. For the pandemic and post-pandemic periods, terms of change in level and trend were included. A 5% significance level (p<0.05) was adopted. Additional details about the modeling are described in the Supplementary Material.

### Segmented regression model for population rates of visits

Furthermore, models were estimated using the monthly number of mental disorder-related visits as a dependent variable, including as offset the logarithm of the resident population estimated for each region, according to data from the 2022 Demographic Census of the Brazilian Institute of Geography and Statistics (IBGE). Models were also adjusted having as dependent variable the total monthly number of visits in PHC. These models maintained the segmented regression structure adopted in the analysis by proportional rates, in order to assess whether the findings could be influenced by variations in the total volume of PHC services over the period.

### Ethical aspects

As this is a study with aggregating, public domain data, and without identification of individuals, the study is exempted from approval by the Research Ethics Committee, according to Resolution No. 510/2016, of the National Health Council (*Conselho Nacional de Saúde* - CNS).

## Data Availability Statement:

The data that support the results derive from public sources (SISAB/DATASUS). The dataset integrated and processed by the authors is available upon request to the corresponding author.

## RESULTS

### Descriptive analysis

Between January 2018 and December 2023, 38.7 million visits were registered with primary diagnosis of mental disorder in PHC. The median of monthly proportions of these visits, nationally, increased from 1.90% in the pre-pandemic period to 2.88% of the total PHC visits in the post-pandemic period (Table 1).

**Table 1.** Number and proportion of mental disorder-related visits in Primary Health Care (Brazil and macroregions, 2018-2023; monthly median interquartile range).

Geographic unit	Total (2018 to 2023)		Pre-pandemic		Social distancing		Pandemic		Post-pandemic	
	Visits	% PHC*	Monthly median (IQR)	% PHC*	Monthly median (IQR)	% PHC*	Monthly median (IQR)	% PHC*	Monthly median (IQR)	% PHC*
Brazil	38795399	2.46	360686.5 (78590.25)	1.90	344455.0 (48529.0)	2.78	509752.0 (93478.0)	2.51	789196.0 (204127.0)	2.88
Midwest	3029967	2.61	29369.5 (6284.5)	2.25	31097.0 (3801.0)	2.98	38171.0 (7461.0)	2.58	62179.0 (13875.0)	2.97
Northeast	7761815	1.99	62092.0 (14715.5)	1.15	65036.0 (5337.5)	2.09	100095.0 (26585.0)	2.06	174538.0 (42671.0)	2.68
North	1470280	1.44	13632.0 (2939.75)	1.08	13058.0 (1613.0)	1.52	19523.0 (5453.0)	1.32	30268.0 (7962.0)	1.79
Southeast	17269462	2.71	161956.5 (42904.25)	2.10	148107.0 (25140.0)	3.08	233185.0 (33709.0)	2.83	353048.0 (86025.0)	3.05
South	9263875	2.79	95571.0 (15018.5)	2.65	87157.0 (12637.5)	3.37	118612.0 (26835.0)	2.76	172807.0 (40856.0)	2.96

*proportion between mental disorder-related visits and total visits in Primary Health Care.

PHC: Primary Health Care; IQR: interquartile range.

Source: Prepared by the authors.

Regionally, the highest proportions were verified in the South and the Southeast throughout the period, while lower levels were identified in the North and the Northeast, with more accentuated growth in the latter.

#### Analysis of segmented regression models for proportional rates of visits

In Table 2, we show the estimates of the monthly trend in the pre-pandemic period and the effects of changes in level and trend associated with the subsequent phases. In the social distancing, pandemic, and post-pandemic periods, the exponential coefficients (exp(β)) represent multiplying ratios in the proportion of mental disorder-related visits, with the pre-pandemic period as a reference.

**Table 2.** Estimates of change in level and trend in the proportion of mental disorder-related visits in Primary Health Care, associated with the pandemic phases.

Stratification	Change effect	Pre-pandemic		Social distancing		Pandemic		Post-pandemic	
		exp(β)	95%CI	exp(β)	95%CI	exp(β)	95%CI	exp(β)	95%CI
Geographic units									
Brazil	Level			1.241	1.149-1.339	1.079	1.005-1.158	0.855	0.760-0.961
	Trend	1.011	1.008-1.014			0.989	0.984-0.994	1.001	0.996-1.006
Midwest	Level			1.179	1.065-1.306	0.977	0.885-1.079	0.877	0.744-1.033
	Trend	1.008	1.004-1.012			0.994	0.987-1.001	0.999	0.992-1.006
Northeast	Level			1.404	1.271-1.551	1.263	1.142-1.398	1.032	0.871-1.223
	Trend	1.016	1.012-1.020			0.989	0.982-0.996	1.001	0.994-1.008
North	Level			1.186	1.079-1.303	0.918	0.835-1.009	0.980	0.839-1.145
	Trend	1.011	1.007-1.015			1.003	0.997-1.010	1.004	0.998-1.011
Southeast	Level			1.198	1.104-1.300	1.059	0.980-1.144	0.779	0.685-0.885
	Trend	1.013	1.010-1.016			0.987	0.981-0.992	0.996	0.991-1.002
South	Level			1.189	1.089-1.298	1.053	0.967-1.146	0.809	0.703-0.930
	Trend	1.005	1.002-1.008			0.988	0.982-0.993	1.007	1.001-1.013
Diagnostic groups (Brazil)									
Organic Mental Disorders (F00-F09)	Level			1.225	1.127-1.332	1.132	1.046-1.225	0.896	0.787-1.021
	Trend	1.008	1.005-1.011			0.987	0.982-0.993	0.997	0.992-1.003
Mental and Behavioral Disorders Due to Psychoactive Substances (F10-F19)	Level			1.246	1.148-1.352	1.181	1.096-1.272	0.898	0.795-1.015
	Trend	1.009	1.006-1.011			0.984	0.979-0.989	1.007	1.002-1.012
Schizophrenia and Delusional Disorders (F20-F29)	Level			1.214	1.114-1.323	1.041	0.954-1.136	0.785	0.678-0.908
	Trend	1.007	1.003-1.010			0.987	0.981-0.993	1.001	0.995-1.007
Mood Disorders (F30-F39)	Level			1.279	1.183-1.384	1.057	0.985-1.135	0.736	0.655-0.828
	Trend	1.008	1.005-1.011			0.983	0.978-0.988	0.994	0.989-0.999
Anxiety Disorders (F40-F48)	Level			1.366	1.254-1.488	1.195	1.101-1.298	0.946	0.825-1.085
	Trend	1.015	1.011-1.018			0.989	0.983-0.995	1.000	0.994-1.005
Physiological Disorders (F50-F59)	Level			1.425	1.323-1.535	1.279	1.194-1.370	0.685	0.611-0.767
	Trend	1.011	1.008-1.014			0.971	0.966-0.976	0.992	0.987-0.996
Personality Disorders (F60-F69)	Level			1.003	0.912-1.103	1.023	0.938-1.117	0.635	0.548-0.734
	Trend	1.021	1.018-1.025			0.982	0.976-0.988	0.991	0.986-0.997
Intellectual Disabilities (F70-F79)	Level			0.794	0.709-0.889	0.817	0.738-0.906	0.862	0.728-1.022
	Trend	1.007	1.003-1.011			1.006	0.999-1.014	1.006	0.998-1.013
Disorders of Psychological Development (F80-F89)	Level			0.395	0.336-0.465	0.565	0.483-0.661	0.863	0.666-1.119
	Trend	1.015	1.009-1.021			1.025	1.014-1.036	1.011	1.000-1.022
Behavioral and Emotional Disorders with onset usually occurring in childhood and adolescence (F90-F98)	Level			0.845	0.749-0.953	0.815	0.725-0.916	1.005	0.828-1.220
	Trend	1.002	0.997-1.007			1.010	1.002-1.019	1.021	1.013-1.029
Mental Disorder, not otherwise specified (F99)	Level			1.569	1.403-1.754	1.133	0.999-1.284	0.507	0.409-0.629
	Trend	1.026	1.021-1.031			0.964	0.956-0.973	0.985	0.976-0.994

exp(β): rate ratio estimated by the model; 95%CI: 95% confidence interval.

Values in bold indicate statistical significance (p<0.05).

Source: Prepared by the authors.

In Brazil, we verified a modest monthly growth during the pre-pandemic period of the proportion of mental disorder-related visits (exp(β)_Trend_=1.011; 95% Confidence Interval [95%CI] 1.008-1.014). During social distancing, there was an immediate increase in the level of the series (exp(β)_Level_=1.241; 95%CI 1.149-1.339). During the rest of the pandemic, the level remained above the counterfactual (exp(β)_Level_=1.079; 95%CI 1.005-1.158), with a decrease in the monthly trend (exp(β)_Trend_=0.989; 95%CI 0.984-0.994). In the post-pandemic period, we observed a lower level than the counterfactual (exp(β)_Level_=0.855; 95%CI 0.760-0.961), with no significant change in the monthly slope.

The regions followed a general pattern similar to the national’s, with a significant increase in the level in the period of social distancing: the Northeast presented the highest increase (exp(β)_Level_=1.404; 95%CI 1.271-1.551), while the Midwest registered the lowest (exp(β)_Level_=1.179), with intermediate values in the other regions. During the pandemic, only the Northeast maintained a significant increase in level (exp(β)_Level_=1.263; 95%CI 1.142-1.398), while the Southeast and South presented a deceleration of the monthly trend. In the post-pandemic period, the Southeast and South regions presented decreases in level, while the North and Northeast maintained an upward pattern. Regional heterogeneity was noteworthy, with strong statistical evidence for the interaction term (p<0.001).

We verified an abrupt increase in the level of anxiety and mood disorders during social distancing, more pronounced for anxiety disorders (exp(β)_Level_=1.366; 95%CI 1.254-1.488) than for mood disorders (exp(β)_Level_=1.279; 95%CI 1.183-1.384). During the pandemic, a significant increase in the level of anxiety disorders (exp(β)_Level_=1.195; 95%CI 1.101-1.298) remained, in addition to physiological disorders, those related to the use of substances, and organic disorders. Mood disorders did not differ from the counterfactual. In the post-pandemic period, anxiety disorders were stabilized in relation to the counterfactual, while mood disorders remained below the expected level. Developmental disorders and Behavioral and Emotional Disorders with onset usually occurring in childhood and adolescence (ICD-10 F90-F98) presented an initial drop during social distancing and the pandemic periods, with progressive recovery. In the post-pandemic period, ICD-10 F90-F98 disorders stood out for significant acceleration of the trend (exp(β)_Trend_=1.021; 95%CI 1.013-1.029).

#### Analysis of segmented regression models for population rates of visits

**Figure 1.**
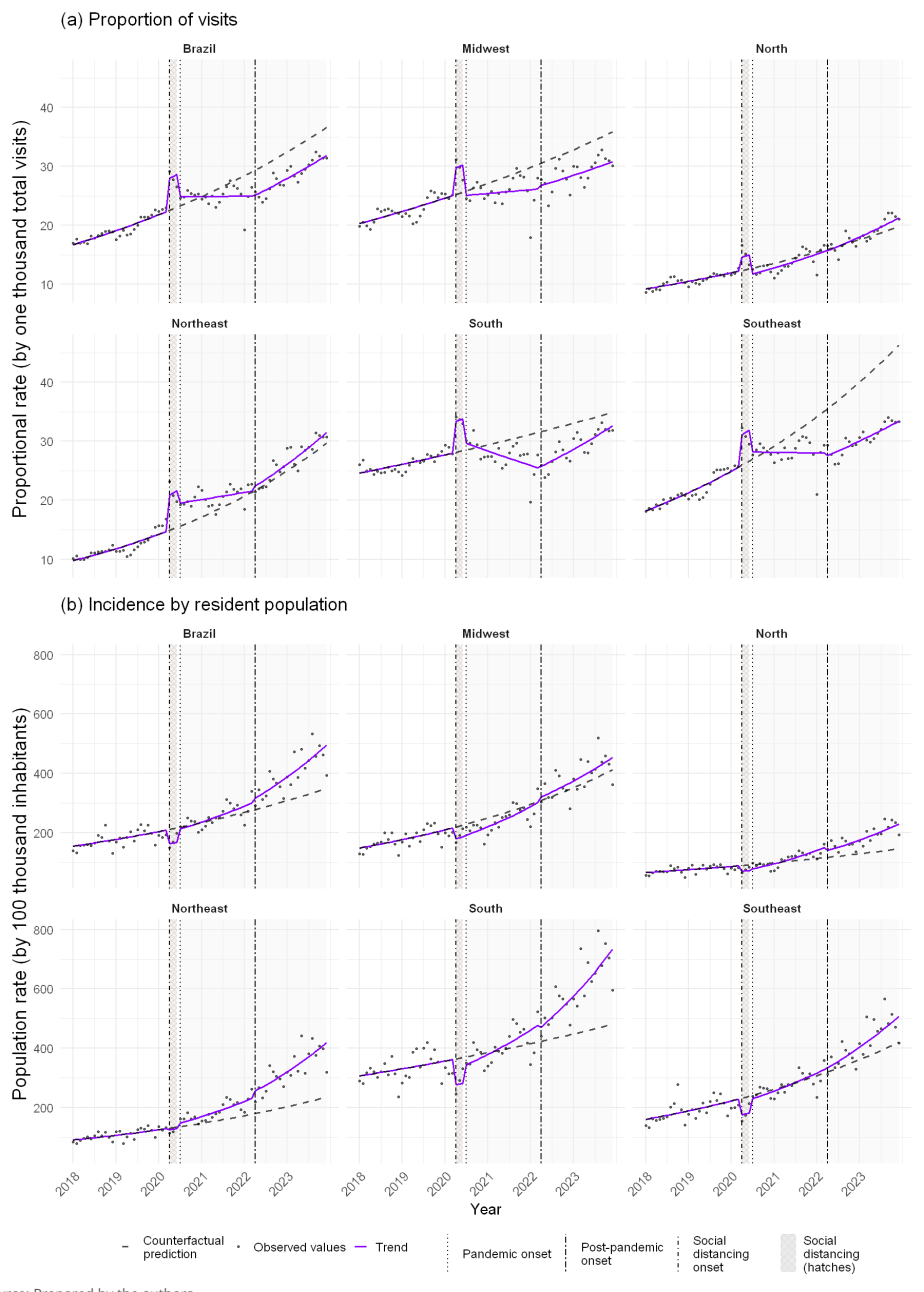
Time series of mental disorder-related visits in Primary Health Care by geographic unit (2018-2023): (a) proportion of visits; (b) incidence by resident population.

Source: Prepared by the authors.

There was a significant increasing trend in the rates of mental disorder-related visits in Brazil before the pandemic (exp(β)_Trend_=1.011; 95%CI 1.010-1.013), while the total PHC rates remained stable, with a similar pattern in the macroregions (Table 3). In the period of social distancing, there was a significant reduction in the total PHC rates in Brazil (exp(β)_Level_=0.609; 95%CI 0.544-0.682), which was also observed in the other macroregions, while the rates of mental disorder-related visits presented a slight decrease. During the pandemic, mental health rates remained stable, with gradual recovery of total visits. In the post-pandemic period, we verified an increase in the rates of mental disorder-related visits in some regions, especially in the Northeast (exp(β)_Level_=1.409; 95%CI 1.284-1.545), concomitant with the recomposition of total visits (Figure 1).

**Table 3.** Estimates of change in level and trend in the incidence rate of mental disorder-related visits and total visits in Primary Health Care, associated with the pandemic phases.

Stratification	Change effect	Pre-pandemic		Social distancing		Pandemic		Post-pandemic	
		exp(β)	95%CI	exp(β)	95%CI	exp(β)	95%CI	exp(β)	95%CI
Total visits in Primary Health Care									
Brazil	Level			0.609	0.544-0.682	0.889	0.834-0.947	1.318	1.189-1.461
	Trend	1.000	0.998-1.003			1.017	1.012-1.022	1.009	1.004-1.014
Midwest	Level			0.675	0.604-0.753	0.833	0.783-0.887	1.158	1.047-1.280
	Trend	1.007	1.005-1.009			1.014	1.010-1.019	1.002	0.998-1.007
Northeast	Level			0.581	0.522-0.646	0.831	0.787-0.877	1.239	1.132-1.356
	Trend	1.000	0.997-1.002			1.017	1.013-1.021	1.008	1.004-1.012
North	Level			0.623	0.555-0.699	0.929	0.874-0.987	1.190	1.076-1.316
	Trend	1.001	0.999-1.004			1.014	1.009-1.018	1.007	1.002-1.012
Southeast	Level			0.644	0.545-0.761	0.908	0.786-1.050	1.354	1.065-1.721
	Trend	1.000	0.994-1.006			1.017	1.007-1.028	1.011	1.001-1.021
South	Level			0.593	0.534-0.658	0.885	0.834-0.938	1.337	1.216-1.469
	Trend	1.002	1.000-1.004			1.019	1.015-1.024	1.008	1.004-1.012
Mental disorder-related visits (ICD-10 F00-F99)									
Brazil	Level			0.771	0.707-0.840	0.971	0.930-1.014	1.130	1.051-1.214
	Trend	1.011	1.010-1.013			1.005	1.002-1.009	1.011	1.008-1.014
Midwest	Level			0.824	0.766-0.887	0.832	0.804-0.862	1.034	0.974-1.097
	Trend	1.014	1.013-1.016			1.008	1.006-1.011	1.003	1.000-1.006
Northeast	Level			0.964	0.866-1.073	1.085	1.026-1.147	1.409	1.284-1.545
	Trend	1.013	1.011-1.016			1.009	1.004-1.013	1.011	1.007-1.015
North	Level			0.788	0.710-0.875	0.834	0.791-0.880	1.186	1.086-1.296
	Trend	1.011	1.009-1.013			1.021	1.017-1.025	1.013	1.009-1.017
Southeast	Level			0.763	0.646-0.901	0.952	0.822-1.102	1.036	0.813-1.320
	Trend	1.014	1.008-1.019			1.004	0.994-1.014	1.007	0.997-1.018
South	Level			0.763	0.661-0.882	0.918	0.809-1.041	1.097	0.890-1.351
	Trend	1.006	1.001-1.011			1.010	1.001-1.019	1.016	1.007-1.025
Diagnostic groups (Brazil)									
Organic Mental Disorders (F00-F09)	Level			0.778	0.715-0.846	1.026	0.984-1.069	1.208	1.126-1.295
	Trend	1.008	1.007-1.010			1.004	1.000-1.007	1.007	1.004-1.010
Mental and Behavioral Disorders Due to Psychoactive Substances (F10-F19)	Level			0.814	0.716-0.925	1.077	1.000-1.159	1.250	1.110-1.408
	Trend	1.008	1.005-1.011			1.001	0.996-1.006	1.017	1.012-1.022
Schizophrenia and Delusional Disorders (F20-F29)	Level			0.796	0.704-0.900	0.927	0.857-1.002	1.079	0.954-1.220
	Trend	1.006	1.003-1.009			1.006	1.001-1.011	1.011	1.006-1.017
Mood Disorders (F30-F39)	Level			0.789	0.725-0.858	0.938	0.899-0.979	0.957	0.891-1.028
	Trend	1.009	1.007-1.011			0.999	0.996-1.003	1.003	1.000-1.007
Anxiety Disorders (F40-F48)	Level			0.877	0.803-0.959	1.080	1.034-1.129	1.265	1.174-1.363
	Trend	1.015	1.013-1.017			1.006	1.002-1.009	1.010	1.007-1.013
Physiological Disorders (F50-F59)	Level			0.896	0.822-0.977	1.146	1.097-1.197	0.905	0.841-0.974
	Trend	1.011	1.010-1.013			0.987	0.984-0.991	1.001	0.998-1.005
Personality Disorders (F60-F69)	Level			0.637	0.538-0.754	0.904	0.779-1.048	0.824	0.644-1.054
	Trend	1.022	1.016-1.028			1.000	0.990-1.011	1.001	0.991-1.011
Intellectual Disabilities (F70-F79)	Level			0.466	0.408-0.531	0.745	0.692-0.802	1.125	0.999-1.268
	Trend	1.007	1.004-1.010			1.023	1.018-1.029	1.017	1.012-1.023
Disorders of Psychological Development (F80-F89)	Level			0.241	0.192-0.303	0.499	0.404-0.615	1.088	0.769-1.539
	Trend	1.016	1.007-1.024			1.043	1.028-1.059	1.021	1.007-1.036
Behavioral and Emotional Disorders with onset usually occurring in childhood and adolescence (F90-F98)	Level			0.521	0.433-0.627	0.713	0.605-0.839	1.276	0.974-1.672
	Trend	1.003	0.997-1.010			1.029	1.017-1.040	1.030	1.019-1.042
Mental Disorder, not otherwise specified (F99)	Level			1.093	0.989-1.209	1.005	0.930-1.085	0.690	0.606-0.786
	Trend	1.026	1.023-1.029			0.983	0.978-0.988	0.994	0.988-0.999

exp(β): rate ratio estimated by the model; 95%CI: 95% confidence interval.

Values in bold indicate statistical significance (p<0.05).

Source: Prepared by the authors.

**Figure 2.**
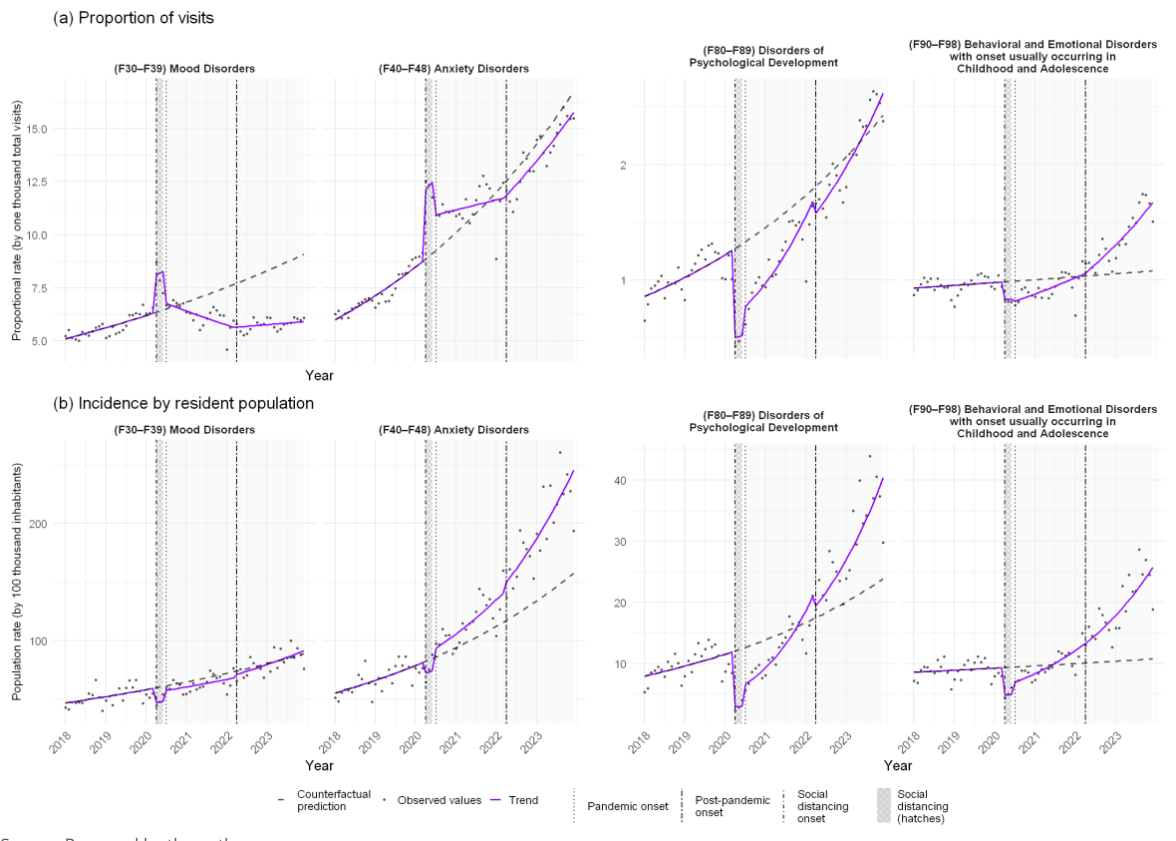
Time series of visits in Primary Health Care for selected diagnostic groups, Brazil (2018-2023): (a) proportion of visits; (b) incidence by resident population.

Source: Prepared by the authors.

In the analysis by diagnostic groups, we observed an increase in mental disorder-related visits, with an increase during the pandemic and post-pandemic periods, followed by an increase in the temporal trend. In the post-pandemic period, we verified increases in trends for visits related to ICD-10 F90-F98 disorders and disorders of psychological development (Figure 2). Complete results can be found in the Supplementary Material.

## DISCUSSION

In this study, we identified a progressive increase in the share of mental disorder-related visits in PHC in Brazil, a trend already present before the pandemic. The onset of the Covid-19 pandemic and the period of social distancing resulted in an abrupt change in the composition of the care services, with a proportional increase in mental health records. We observed regional heterogeneity, with more pronounced growth in the Northeast. Among the diagnostic groups, we highlight the increase in anxiety disorders and the recent acceleration of the diagnosis of developmental disorders and those occurring in childhood.

During social distancing, we verified significant decrease in the total number of PHC visits, reducing the denominator of the analyzed ratio. Thus, the proportional increase in mental disorder-related visits may reflect not only increased demand, but also more intense retraction of other types of care. According to the complementary analysis by incidence rates, mental health-related visits have slightly and relatively decreased, indicating differential maintenance of care, although the combination between demand increase and supply restriction should be considered.

The temporal segmentation allowed us to identify that the initial shock of the pandemic and social distancing resulted in an abrupt increase in the proportion of visits, followed by a deceleration of the trend. In the post-pandemic period, we observed a resumption of previous monthly growth, although with an average level lower than the estimated counterfactual. As per the analysis by incidence, this result partly reflects the faster recomposition of the total volume of PHC visits in relation to mental disorder-related visits after the initial shock of the pandemic. We highlight the new acceleration pattern observed in ICD-10 F90-F98 disorders, indicating possible recent reconfiguration in the profile of care demand.

Regionally, we verified a more pronounced growth in mental disorder-related visits in the Northeast throughout the analyzed period. This pattern may reflect the structural centrality of PHC in the organization of regional care, marked by broad coverage of the Family Health Strategy and greater dependence of the population on the SUS, in contrast to the greater weight of the private sector in the Southeast and the historical constraints of the North-related to the availability of professionals and geographic challenges^16^. Differences in RAPS regional organization can also influence this pattern. In contexts where specialized services, such as CAPS, play a greater role in serving as the gateway or continuity of care, part of the demand for mental health can focus on these services, reducing their relative expression in PHC. In the post-pandemic period, the growth observed in the Northeast region suggests possible reorganization of the care flow and greater prominence of PHC in mental health care.

As for the diagnostic groups, we identified two distinct profiles of response to the pandemic. On the one hand, for some diagnoses, which include both CMD and severe mental disorders, we verified an abrupt increase in proportion during social distancing, followed by a deceleration of the trend, a pattern consistent with evidence on the intensification of anxiety and depression symptoms in contexts of social isolation^10^ ^,^ ^14^ ^,^ ^17^ ^,^ ^18^. On the other hand, for disorders predominantly diagnosed in childhood or individuals who depend on caregivers for accessing the services, we observed a sharp decline during the period of social distancing, with subsequent gradual recovery, suggesting transitional barriers to access during the pandemic-a phenomenon already documented in other countries for, for example, patients with Autistic Spectrum Disorder (ASD) and their relatives^19^.

Among these groups, mood and anxiety disorders represent the majority of PHC visits and showed an increase in proportion in the early months of the pandemic, a pattern also observed in other countries^13^. Although mood and anxiety disorders presented similar levels at the beginning of the series, there was a disproportionate increase in anxiety disorders over the years, especially after 2020.

The analysis by population rates provides an additional perspective: while some diagnostic groups showed evident changes in the incidence of visits, these changes did not always reflect in equivalent changes in proportional rates. This mismatch suggests that part of the observed increase may result from the expansion of access to health care. However, when comparing anxiety and mood disorders, we verified different patterns in the incidence model: although both represent the most frequent groups, mood disorders followed more closely the estimated counterfactual pattern, while anxiety disorders remained above this counterfactual, suggesting that the expansion observed in the incidence of anxiety disorder-related visits can hardly be attributed only to the expansion of access to services. Nonetheless, it should be noted that anxiety and mood disorders have high comorbidity^20^; in addition, mixed anxiety-depressive disorders are the most commonly found in PHC^21^, possibly leading to diagnostic overlaps.

Another relevant aspect was the increase in visits related to disorders of psychological development and ICD-10 F90-F98 disorders in the post-pandemic period. Developmental disorders, especially ASD, have become more frequent over time at a global level^22^. Although there are different explanations for this increase, a central hypothesis is the expansion of diagnostic criteria, with the notion of spectrum and a more inclusive clinical threshold^23^; a phenomenon that can also explain the higher prevalence of Attention-Deficit/Hyperactivity Disorder (ADHD)^24^. Institutional and normative factors, such as linking social rights to formal diagnoses, may have contributed to the expansion of the records, although this possibility could not be directly tested in this study. Thus, it is possible that part of the observed growth reflects changes in diagnostic practices, not necessarily changes in demand.

The low proportion of visits related to the use of psychoactive substances throughout the period is noteworthy. Although there was a slight increase in the post-pandemic period, the proportional share of these diseases in PHC remains reduced. This pattern may be associated with the centralization of care in specialized services (such as Psychosocial Care Centers for Alcohol and Other Drugs), the stigmatization of users, and the persistence of the logic of hospitalization as the main therapeutic route^25^ ^,^ ^26^. Furthermore, the possible low incorporation of systematic screening strategies and brief interventions in PHC can contribute to underdetection in the early stages, a hypothesis that should be further investigated. Considering the high prevalence and the impact of these disorders in Brazil^27^, the findings suggest that PHC is underutilized in the management of these disorders.

We suggest further investigations focusing on the aspect of diagnostic or record practices, especially to corroborate the findings related to increased visits related to developmental disorders, ICD-10 F90-F98, and anxiety disorders. Moreover, we recommend additional investigations with a specific geographic focus on the regions of Brazil that consider both local health policies and their impacts on mental health care, as well as geographic specificities of demand and access. In addition, analyses with comparisons with other RAPS services, identifying whether PHC has been absorbing the repressed demand of these services, especially CAPS, should be carried out. Finally, investigations assessing the impact of implementing care for mental disorders in PHC on outcomes, such as hospitalization or death rates, are instrumental in supporting decisions and prioritizing investments in public mental health management.

Despite the robustness of the study, we highlight the possible inflation of the pre-pandemic trend as a limitation due to the ongoing implementation of SISAB at the time (with probable stabilization as of 2019)^28^, which may have artificially increased the counterfactual trend, as municipalities with different profiles and registration practices were gradually incorporated into the system. In addition, the statistical model did not include variables, such as demographic, socioeconomic, or local policy factors, which limits attributing isolated causality to the pandemic event. This is an ecological study, in such a way that the results should be interpreted only at the population level.

In this study, we demonstrated that mental disorder-related visits had been progressively increasing in share in Brazilian PHC before the pandemic, reflecting a structural process of integrating mental health at this level of care. The initial period of social distancing was marked by a change in the composition of the care services, mainly due to the more intense retraction of the total volume of PHC, although mental health-related visits have presented a relatively lower reduction. In the subsequent period, we observed a resumption of the previous pattern, in a heterogeneous way between macroregions, with emphasis on the consolidation of anxiety disorders as the most frequent diagnostic group. Overall, the findings suggest that the pandemic acted as a situational shock in a structural trend already underway, rather than as a transforming element of the organization of mental health care in PHC.

## Supplementary Material



## References

[1] Gonçalves DA, Mari JJ, Bower P, Gask L, Dowrick C, Tófoli LF (2014). Brazilian multicentre study of common mental disorders in primary care: rates and related social and demographic factors. Cad Saude Publica.

[2] Häfele V, Nobre ML, Siqueira FV (2023). Prevalência de transtornos mentais e fatores associados em usuários da Atenção Primária. Cad Saude Colet.

[3] Gonçalves DM, Kapczinski F (2008). Prevalência de transtornos mentais em indivíduos de uma unidade de referência para Programa Saúde da Família em Santa Cruz do Sul, Rio Grande do Sul, Brasil. Cad Saude Publica.

[4] Pan American Health Organization (2021). The burden of mental disorders in the Region of the Americas, 2000-2019.

[5] Arias D, Saxena S, Verguet S (2022). Quantifying the global burden of mental disorders and their economic value. EClinicalMedicine.

[6] Mnookin S (2016). Out of the shadows: making mental health a global development priority.

[7] Mari JJ, Thornicroft G (2010). Princípios que deveriam nortear as políticas de saúde mental em países de baixa e média rendas. Rev Bras Psiquiatr.

[8] Hanna F, Barbui C, Dua T, Lora A, van Regteren Alterna M, Saxena S (2018). Global mental health: how are we doing?. World Psychiatry.

[9] Campos RO, Santos DVD, Diaz AV, Emerich B, Trape T, Gama CAP (2020). Estudos de Saúde Mental publicados nos últimos 25 anos na Revista Ciência & Saúde Coletiva. Cien Saude Colet.

[10] Santomauro DF, Mantilla Herrera AM, Shadid J, Zheng P, Ashbaugh C (2021). Global prevalence and burden of depressive and anxiety disorders in 204 countries and territories in 2020 due to the COVID-19 pandemic. Lancet.

[11] Filip R, Puscaselu RG, Anchidin-Norocel L, Dimian M, Savage WK (2022). Global challenges to public health care systems during the COVID-19 pandemic: a review of pandemic measures and problems. J Pers Med.

[12] Machado AV, Ferreira WE, Vitória MAA, Magalhães HM (2023). COVID-19 e os sistemas de saúde do Brasil e do mundo: repercussões das condições de trabalho e de saúde dos profissionais de saúde. Cien Saude Colet.

[13] Silva-Valencia J, Lapadula C, Westfall JM, Gaona G, Lusignan S, Kristianssin RS (2024). Effect of the COVID-19 pandemic on mental health visits in primary care: an interrupted time series analysis from nine INTRePID countries. eClinicalMedicine.

[14] Murray J, Bauer A, Mola CL, Martins RC, Blumenberg C, Esposti MD (2023). Child and maternal mental health before and during the COVID-19 pandemic: longitudinal social inequalities in a Brazilian Birth Cohort. J Am Acad Child Adolesc Psychiatry.

[15] Bernal JL, Cummins S, Gasparrini A (2017). Interrupted time series regression for the evaluation of public health interventions: a tutorial. Int J Epidemiol.

[16] Andrade MV, Coelho AQ, Xavier MX, Carvalho LR, Atun R, Castro MC (2018). Transition to universal primary health care coverage in Brazil: Analysis of uptake and expansion patterns of Brazil’s Family Health Strategy (1998-2012). PLoS One.

[17] Chang JJ, Ji Y, Li YH, Pan HF, Su PY (2021). Prevalence of anxiety symptom and depressive symptom among college students during COVID-19 pandemic: a meta-analysis. J Affect Disord.

[18] Smith GE, Harcourt SE, Hoang U, Lemanska A, Elliot AJ, Morbey RA (2022). Mental health presentations across health care settings during the first 9 months of the COVID-19 pandemic in England: retrospective observational study. JMIR Public Health Surveill.

[19] Baweja R, Brown SL, Edwards EM, Murray MJ (2022). COVID-19 pandemic and impact on patients with autism spectrum disorder. J Autism Dev Disord.

[20] Lamers F, van Oppen P, Comijs HC, Smit JH, Spinhoven P, van Balkom AJLM (2011). Comorbidity patterns of anxiety and depressive disorders in a large cohort study: The Netherlands Study of Depression and Anxiety (NESDA). J Clin Psychiatry.

[21] Choi KW, Kim YK, Jeon HJ (2020). Comorbid anxiety and depression: clinical and conceptual consideration and transdiagnostic treatment. Adv Exp Med Biol.

[22] Zeidan J, Fombonne E, Scorah J, Ibrahim A, Durkin MS, Saxena S (2022). Global prevalence of autism: a systematic review update. Autism Res.

[23] Mottron L, Bzdok D (2020). Autism spectrum heterogeneity: fact or artifact?. Mol Psychiatry.

[24] Polanczyk G V., Willcutt EG, Salum GA, Kieling C, Rohde LA (2014). ADHD prevalence estimates across three decades: An updated systematic review and meta-regression analysis. Int J Epidemiol.

[25] Paula ML, Jorge MSB, Vasconcelos MGF, Albuquerque RA (2014). Assistência ao usuário de drogas na atenção primária à saúde. Psicol Estud.

[26] Araujo ACC (2013). Atenção Primária e dependência química: contribuições do matriciamento em saúde mental. Saude Debate.

[27] Bonadiman CSC, Passos VMA, Mooney M, Naghavi M, Melo APS (2017). A carga dos transtornos mentais e decorrentes do uso de substâncias psicoativas no Brasil: Estudo de Carga Global de Doença, 1990 e 2015. Rev Bras Epidemiol.

[28] Barros RD, Silva LA, Souza LEPF (2024). Avaliação do impacto da implantação do novo sistema de informações da atenção primária à saúde nos registros de atendimentos e visitas domiciliares no Brasil. Cad Saude Publica.

