# Supplementary material for: Trends in mental health care in Brazilian Primary Health Care before, during, and after COVID-19: an ecological interrupted time-series study (2018-2023)
Source: Rev Bras Epidemiol. 2026 Jul 31;29:e260037. doi: 10.1590/1980-549720260037 (PMC13427217; doi:10.1590/1980-549720260037)
Supplement: Supplementary file 1 [file 1980-5497-rbepid-29-e260037-sppl.pdf]

Sumário

1. Detalhes da Modelagem Estatística 2

   1.1 Especificação do modelo GLMM com ligação logarítmica e distribuição binomial negativa 2

   1.2 Interpretação dos Coeficientes 3

   1.3 Modelo de incidência populacional 4

   1.4 Estrutura de Correlação 4

   1.5 Estimativa de Sobredispersão (θ) 4

   1.6 Comparações regionais através de teste de razão de verossimilhança 5

2. Resultados Adicionais 7

   2.1 Tabela suplementar da análise descritiva para todos os grupos de transtornos – Brasil (2018-2023) 7

   2.2 Gráfico suplementar dos resultados da análise de séries temporais interrompidas para todos os grupos de transtornos – Brasil (2018-2023): (a) alta magnitude, (b) baixa magnitude - Modelo de Proporção................................. 9

   2.3 Gráfico suplementar dos resultados da análise de séries temporais interrompidas para todos os grupos de transtornos – Brasil (2018-2023): (a) alta magnitude, (b) baixa magnitude - Modelo de Incidência 10

   2.4 Gráfico suplementar dos resultados da análise de séries temporais interrompidas para Atendimentos Totais na APS – Brasil (2018-2023) - Modelo de Incidência 11

   2.5 Tabela suplementar dos resultados do modelo complementar *glmmTMB*, sem interação - (2018-2023) - Modelo de Proporção..................................... 12

   2.6 Tabela suplementar dos resultados do modelo complementar *glmmTMB*, com interação - (2018-2023) - Modelo de Proporção 12

   2.7 Tabela suplementar dos resultados do teste de razão de verossimilhança entre os modelos com ou sem interação 13

3. Detalhes da Raspagem de Dados 14

1. Detalhes da Modelagem Estatística

Para quantificar os efeitos das fases pandêmica (abr/2020–mar/2022) e pós-pandêmica (abr/2022–dez/2023) sobre as taxas proporcionais mensais de atendimentos por transtornos mentais na atenção primária, foram ajustados Modelos Lineares Generalizados Mistos (*GLMM*) com distribuição Binomial Negativa e função de ligação logarítmica, utilizando a função *glmmPQL* dos pacotes **nlme** e **MASS**, na linguagem R (v. 4.5.0).

1.1 Especificação do modelo GLMM com ligação logarítmica e distribuição binomial negativa

$$
\begin{gathered}
Y_{\{it\}} \sim NegBin\left(\mu_{\{it\}}, \theta\right) \\
log\left(\mu_{\{it\}}\right) = log\left(Offset_{\{it\}}\right) + \\
\beta_0 + \\
\beta_1 t\ + \\
\beta_2 DistanciamentoSocialStep_t + \\
\beta_3 PandemiaStep_t + \\
\beta_4 PandemiaTrend_t + \\
\beta_5 PosPandemiaStep_t + \\
\beta_6 PosPandemiaTrend_t + \\
\beta_7 Cos12_t + \\
\beta_8 Sen12_t
\end{gathered}
$$

onde:

- **Y_{it}:** número de atendimentos mensais por transtornos mentais na unidade i, no tempo t;
- **μ_{it}:** valor esperado de Y_{it}, condicional aos preditores;
- **log($Offset_{it}$):** logaritmo do número total de atendimentos na APS no mês t na unidade i, incluído como offset para modelar a taxa de atendimentos por transtornos mentais.
- **t**: tempo contínuo, em meses, desde janeiro de 2018;
- **$DistanciamentoSocialStep_t$**: variável binária, 1 de abril/2020 a junho/2020, 0 nos demais períodos
- **$PandemiaStep_t$:** variável binária, 1 de julho/2020 a março/2022, 0 nos demais períodos;

- **$PandemiaTrend_t$:** variável contínua crescente, inicia em 1 em julho/2020, +1 por mês até março/2022;
- **$PosPandemiaStep_t$:** variável binária, 1 de abril/2022 a dezembro/2023, 0 nos demais períodos;
- **$PosPandemiaTrend_t$:** variável contínua, inicia em 1 em abril/2022, +1 por mês até dezembro/2023;
- **$Cos12_t$** e **$Sen12_t$**: termos harmônicos de uma expansão de Fourier com período de 12 meses incluídos para ajustar sazonalidade anual na série temporal.

Cada série de dados foi rodada separadamente.

1.2 Interpretação dos Coeficientes

- **β_0:** intercepto fixo, refletindo a taxa média de atendimentos no início da série;
- **β_1:** tendência temporal geral pré-pandêmica;
- **β_2:** mudança de nível imediato durante o período de distanciamento social (abril/2020);
- **β_3:** mudança de nível imediato no período pandêmico subsequente (julho/2020);
- **β_4:** mudança na tendência durante o período pandêmico subsequente;
- **β_5:** mudança de nível imediato ao início da fase pós-pandêmica (abril/2022);
- **β_6:** mudança na tendência durante a fase pós-pandêmica.

Importante destacar que os coeficientes associados aos termos de mudança de nível e de tendência na pandemia e pós-pandemia devem ser interpretados em relação ao período pré-pandêmico como referência. Assim, por exemplo, um exp(β) de 1,12 associado à tendência da fase pandêmica ($\exp(\beta_4)$ = 1,12) indica que a tendência mensal de atendimentos por transtornos mentais aumentou em 12% relativamente à tendência pré-existente, e não que a tendência passou diretamente de, por exemplo, 1,02 para 1,12.

1.3 Modelo de incidência populacional

O modelo de incidência populacional seguiu a mesma estrutura do modelo de proporções, apresentado acima, com a exceção de que o *offset* (logaritmo da exposição), neste modelo, foi a população residente na Unidade Geográfica analisada, obtida em consulta ao Censo Populacional IBGE de 2022.

1.4 Estrutura de Correlação

Aplicou-se uma estrutura de correlação temporal *Autoregressive Moving Average* (ARMA), ambas de ordem 1, aplicada à série mensal dentro de cada unidade, para modelar autocorrelação serial nos resíduos. A escolha da ARMA(1,1) foi confirmada pelo ajuste dos resíduos (normalizados) que não restaram significativamente para a maioria das séries. Abaixo, dispomos os gráficos de resíduos (ACF e PACF) da série de transtornos mentais a nível nacional. Gráficos dos resíduos de outras séries podem ser disponibilizados mediante demanda.

Gráfico dos Resíduos Normalizados – Brasil (2018-2023) – Transtornos Mentais (CID F) - Modelo de Proporção

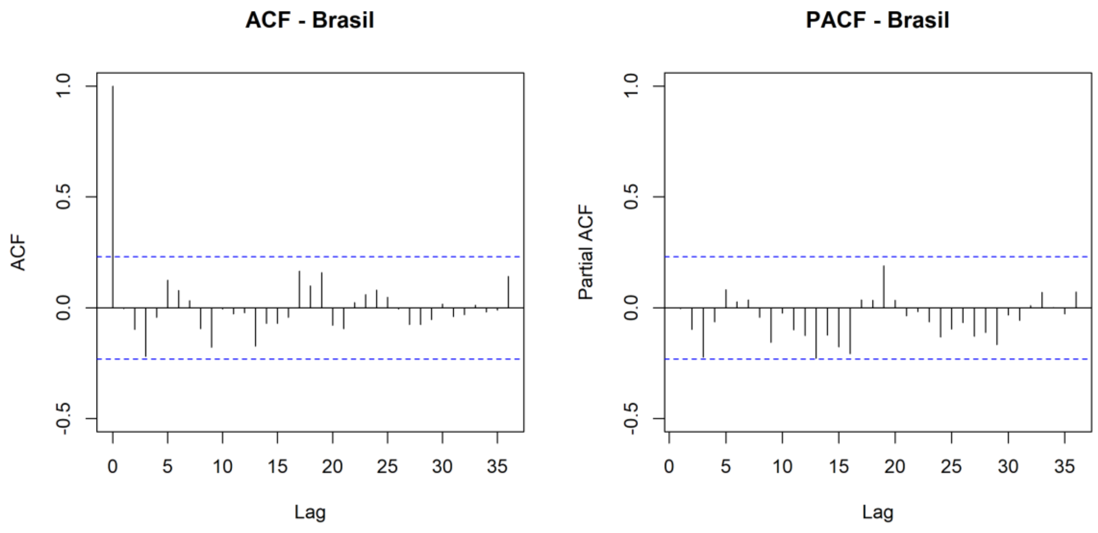

1.5 Estimativa de Sobredispersão (θ)

Devido à presença de sobredispersão nos dados (variância maior que a média), optou-se pelo uso da distribuição Binomial Negativa, parametrizada na forma:

$$Var(Y_i) = \mu_i + \mu_i^2 / \theta$$

em que μ_i representa o valor esperado da resposta no tempo i, e θ é o parâmetro de sobredispersão.

O valor de θ foi estimado por meio do método dos momentos, em duas etapas:

1. Ajustou-se um modelo de Poisson com os mesmos preditores do modelo final.
2. Com base nos valores ajustados (μ_i) e observados (y_i), estimou-se o parâmetro α como:

$$\hat{\alpha} = \Sigma[(y_i - \mu_i)^2 - \mu_i] / \Sigma[\mu_i^2],$$
$$\hat{\theta} = 1 / \hat{\alpha}$$

1.6 Comparações regionais através de teste de razão de verossimilhança

Para avaliar formalmente diferenças regionais na resposta aos períodos temporais segmentados, ajustou-se modelo de regressão binomial negativa utilizando a função *glmmTMB*. O desfecho foi o número mensal de atendimentos por transtornos mentais por unidade geográfica, com inclusão do logaritmo do total de atendimentos na APS como *offset*.

A variável região (Norte, Nordeste, Sudeste, Sul e Centro-Oeste) foi incluída como efeito fixo categórico. Para testar diferenças regionais nas mudanças associadas às fases da pandemia, foram incluídos termos de interação entre região e os indicadores de fase temporal (variáveis de mudança de nível e de tendência definidas no modelo principal).

A sazonalidade anual foi controlada pela inclusão de termos harmônicos de *Fourier* (seno e cosseno com período de 12 meses).

O modelo complementar foi especificado com uma estrutura de correlação AR(1) devido a restrições de implementação do componente MA no *glmmTMB*. Dado que este modelo foi usado exclusivamente para testar formalmente a heterogeneidade regional por meio de termos de interação, esta especificação foi considerada suficiente.

A evidência de heterogeneidade regional foi avaliada pela comparação entre dois modelos aninhados:

1. modelo sem interações, contendo apenas os efeitos principais de região e das variáveis temporais;
2. modelo com interações, incluindo os termos de interação entre região e os indicadores de fase temporal.

A comparação foi realizada por teste de razão de verossimilhança, adotando nível de significância de 5%, via função *ANOVA*.

2. Resultados Adicionais

2.1 Tabela suplementar da análise descritiva para todos os grupos de transtornos – Brasil (2018-2023)

Estratificação	Total (2018 a 2023)		Pré-Pandemia		Distanciamento social		Pandemia		Pós-Pandemia	
	Atendimentos	% APS	Mediana Mensal (IQR)	% APS	Mediana Mensal (IQR)	% APS	Mediana Mensal (IQR)	% APS	Mediana Mensal (IQR)	% APS
Grupos de transtornos (nacionalmente)										
**Transtornos Mentais Orgânicos (F00–F09)**	1739866	0,11%	17541,5 (3249,75)	0,09%	15654,0 (2159,5)	0,13%	23230,0 (3566,0)	0,12%	33913,0 (6226,0)	0,12%
**Transtornos Mentais Devido ao Uso de Substância Psicoativa (F10–F19)**	840859	0,05%	7995,5 (1742,75)	0,04%	7472,0 (1985,5)	0,06%	10940,0 (1169,0)	0,05%	17409,0 (4537,0)	0,06%
**Esquizofrenia e Transtornos Delirantes (F20–F29)**	2412533	0,15%	26452,0 (5146,5)	0,14%	23330,0 (1926,5)	0,19%	31947,0 (3673,0)	0,16%	45370,0 (9507,0)	0,16%
**Transtornos do Humor (F30–F39)**	9309427	0,59%	104484,0 (16298,0)	0,56%	97197,0 (9433,5)	0,78%	127248,0 (15408,0)	0,63%	164052,0 (21466,0)	0,58%
**Transtornos de Ansiedade (F40–F48)**	17339444	1,10%	137534,0 (36142,5)	0,70%	153005,0 (23711,0)	1,23%	232869,0 (46022,0)	1,11%	388668,0 (107456,0)	1,39%

**Transtornos Fisiológicos (F50–F59)**	1211390	0,08%	13166,5 (3274,75)	0,07%	14584,0 (2586,5)	0,12%	17827,0 (3009,0)	0,09%	20781,0 (4193,0)	0,07%
**Transtornos de Personalidade (F60–F69)**	324256	0,02%	2863,0 (940,5)	0,02%	2472,0 (640,5)	0,02%	4606,0 (843,0)	0,02%	6652,0 (1777,0)	0,02%
**Deficiência Intelectual (F70–F79)**	764100	0,05%	7760,5 (1863,75)	0,04%	4380,0 (630,5)	0,04%	8623,0 (3328,0)	0,05%	15713,0 (4011,0)	0,06%
**Transtornos do Desenvolvimento (F80–F89)**	2325489	0,15%	18638,5 (6907,5)	0,10%	5863,0 (1815,5)	0,05%	23674,0 (12919,0)	0,12%	57623,0 (22931,0)	0,21%
**Transtornos do Comportamento e Emocionais da Infância ou Adolescência (F90–F98)**	1742797	0,11%	17728,5 (4820,0)	0,10%	10118,0 (1781,5)	0,08%	18343,0 (5638,0)	0,09%	37709,0 (14066,0)	0,13%
**Transtorno Mental Não Especificado (F99)**	785238	0,05%	6986,5 (2606,5)	0,04%	10380,0 (1858,5)	0,09%	11381,0 (1150,0)	0,05%	15121,0 (3345,0)	0,05%

% APS: proporção entre atendimentos a transtornos mentais e o total de atendimentos na Atenção Primária a Saúde. IQR: intervalo interquartil

2.2 Gráfico suplementar dos resultados da análise de séries temporais interrompidas para todos os grupos de transtornos – Brasil (2018-2023): (a) alta magnitude, (b) baixa magnitude - Modelo de Proporção

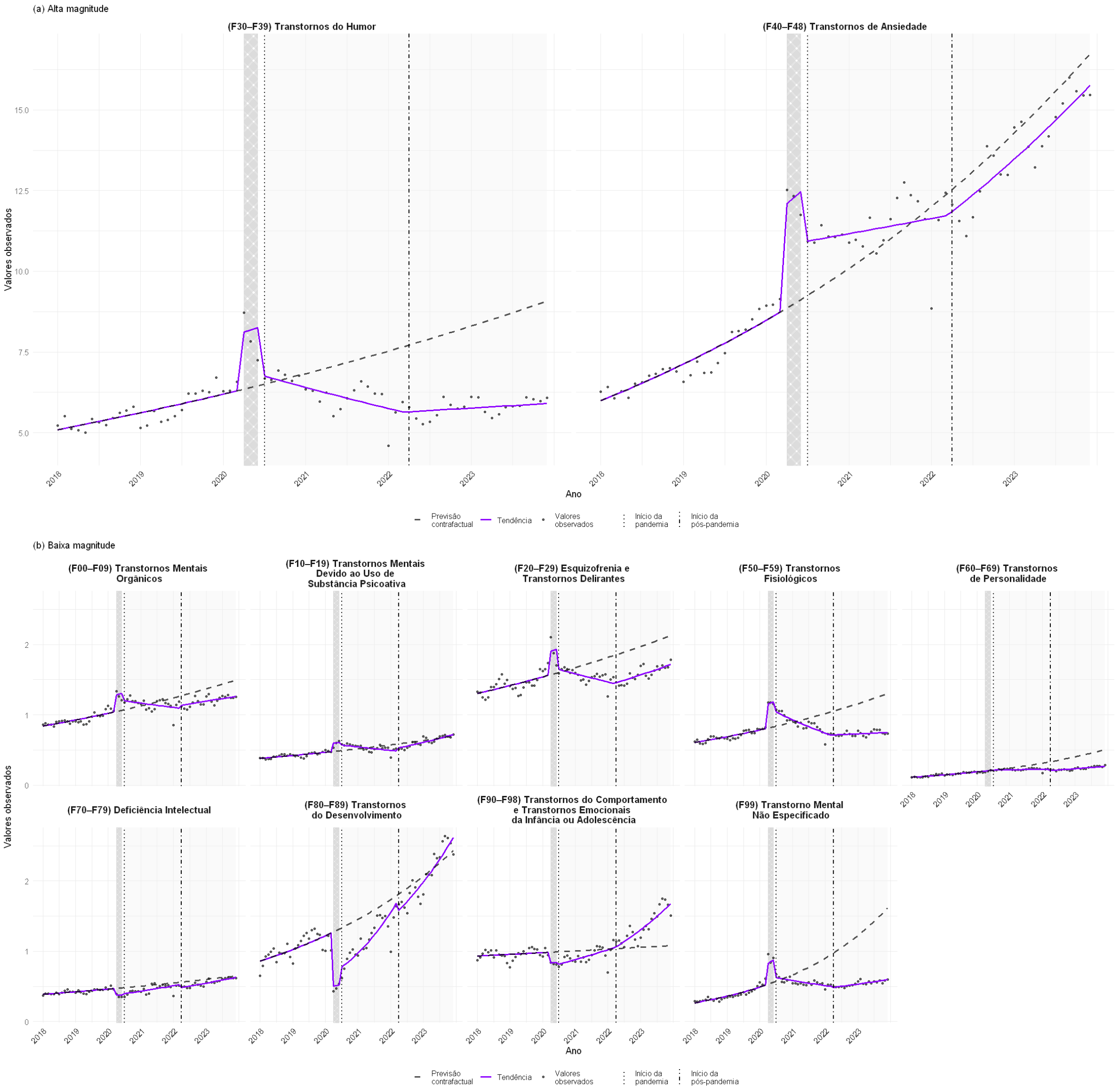

2.3 Gráfico suplementar dos resultados da análise de séries temporais interrompidas para todos os grupos de transtornos – Brasil (2018-2023): (a) alta magnitude, (b) baixa magnitude - Modelo de Incidência

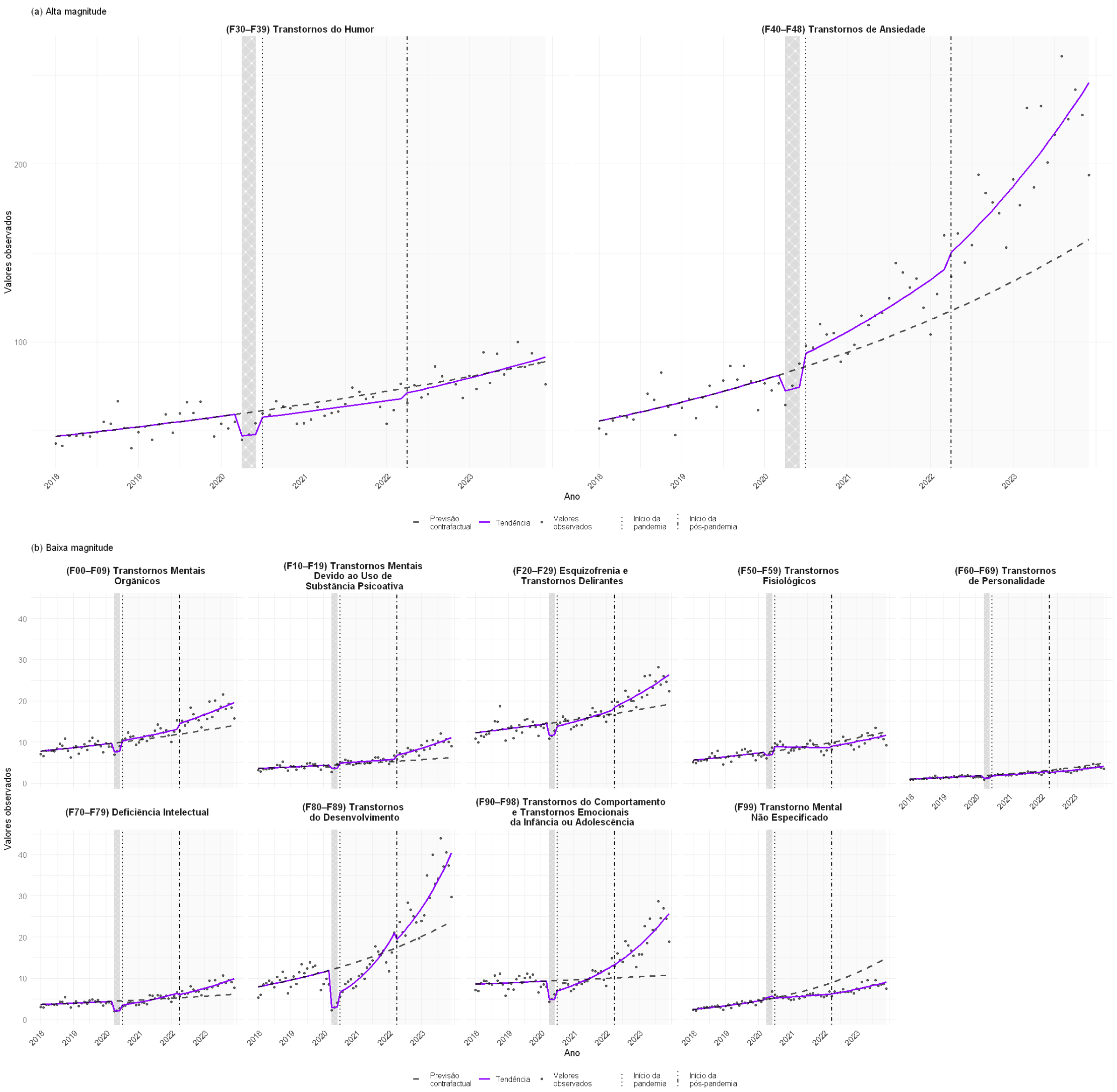

2.4 Gráfico suplementar dos resultados da análise de séries temporais interrompidas para Atendimentos Totais na APS – Brasil (2018-2023) - Modelo de Incidência

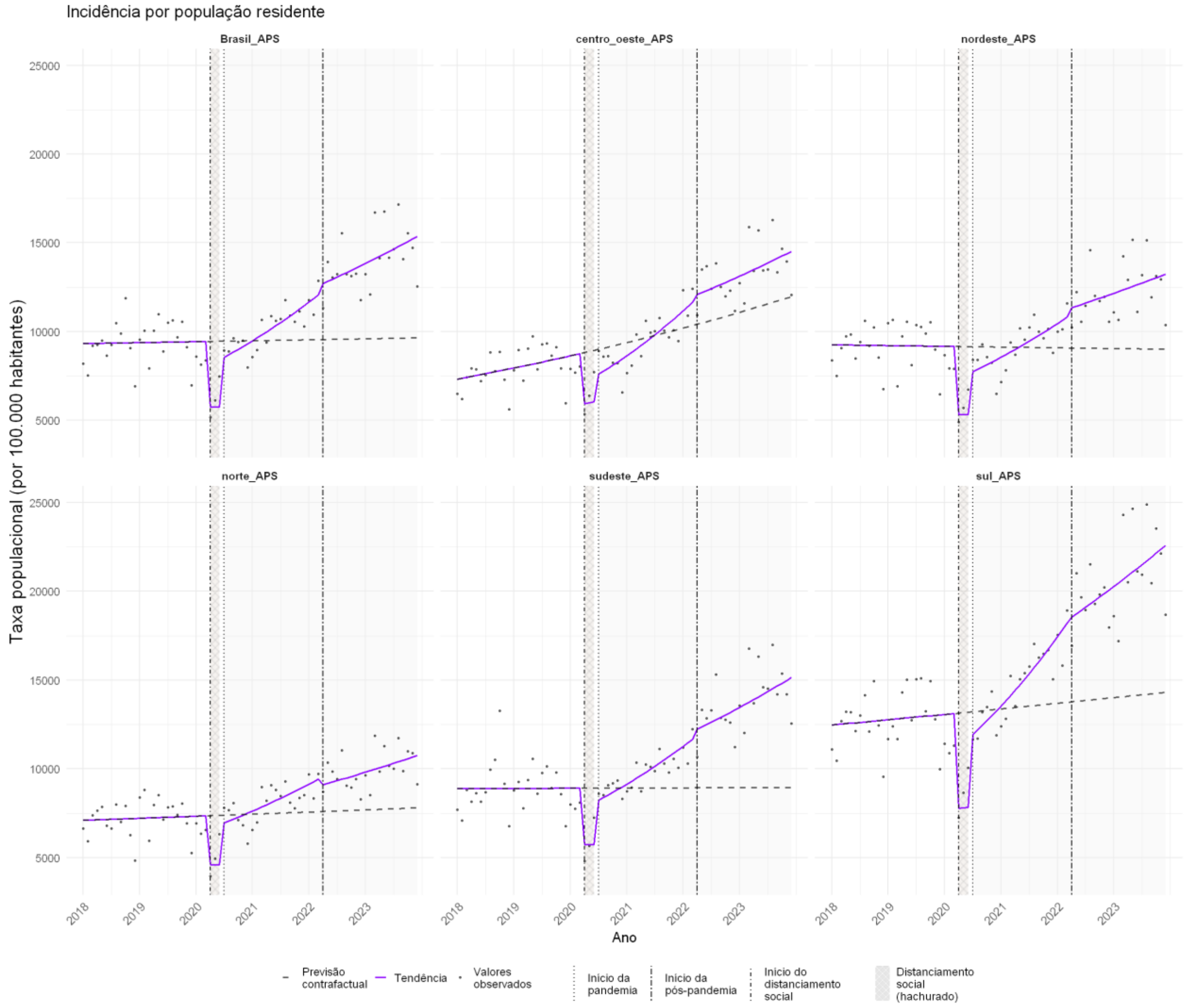

2.5 Tabela suplementar dos resultados do modelo complementar *glmmTMB*, sem interação - (2018-2023) - Modelo de Proporção

Parametro	Estimate	Std, Error	z value	Pr(>|z|)
**(Intercept)**	2,734	0,139	19,707	<0,001
**periodo**	0,010	0,003	3,746	<0,001
**Pandemia_Step**	0,031	0,037	0,833	0,405
**Pandemia_Trend**	-0,006	0,004	-1,332	0,183
**PosPandemia_Step**	-0,090	0,098	-0,912	0,362
**PosPandemia_Trend**	0,001	0,004	0,178	0,859
**Lockdown_Step**	0,199	0,027	7,360	<0,001
**cos1_12**	-0,008	0,006	-1,341	0,180
**sin1_12**	-0,032	0,006	-5,268	<0,001

2.6 Tabela suplementar dos resultados do modelo complementar *glmmTMB*, com interação - (2018-2023) - Modelo de Proporção

Parametro	Estimate	Std, Error	z value	Pr(>|z|)
**(Intercept)**	2,968	0,023	131,711	<0,001
**periodo**	0,010	0,001	10,507	<0,001
**Pandemia_Step**	-0,043	0,046	-0,939	0,348
**regiaonordeste**	-0,623	0,025	-24,652	<0,001
**regiaonorte**	-0,754	0,025	-29,808	<0,001
**regiaosudeste**	-0,042	0,025	-1,648	0,099
**regiaosul**	0,157	0,025	6,209	<0,001
**Pandemia_Trend**	-0,009	0,003	-2,830	0,005
**PosPandemia_Step**	-0,216	0,056	-3,836	<0,001
**PosPandemia_Trend**	-0,003	0,003	-0,880	0,379
**Lockdown_Step**	0,143	0,047	3,050	0,002
**cos1_12**	-0,008	0,006	-1,184	0,237
**sin1_12**	-0,030	0,007	-4,513	<0,001
**Pandemia_Step:regiaonordeste**	0,331	0,061	5,450	<0,001
**Pandemia_Step:regiaonorte**	-0,017	0,061	-0,278	0,781
**Pandemia_Step:regiaosudeste**	0,152	0,061	2,510	0,012
**Pandemia_Step:regiaosul**	0,006	0,061	0,105	0,917
**regiaonordeste:Pandemia_Trend**	0,006	0,004	1,420	0,156
**regiaonorte:Pandemia_Trend**	0,012	0,004	2,746	0,006
**regiaosudeste:Pandemia_Trend**	-0,002	0,004	-0,393	0,694
**regiaosul:Pandemia_Trend**	-0,008	0,004	-1,889	0,059
**regiaonordeste:PosPandemia_Step**	0,425	0,061	6,960	<0,001
**regiaonorte:PosPandemia_Step**	0,213	0,061	3,494	<0,001
**regiaosudeste:PosPandemia_Step**	0,073	0,061	1,192	0,233
**regiaosul:PosPandemia_Step**	-0,199	0,061	-3,265	0,001
**regiaonordeste:PosPandemia_Trend**	0,011	0,004	2,436	0,015
**regiaonorte:PosPandemia_Trend**	0,008	0,004	1,894	0,058
**regiaosudeste:PosPandemia_Trend**	0,002	0,004	0,533	0,594
**regiaosul:PosPandemia_Trend**	0,004	0,004	1,011	0,312
**regiaonordeste:Lockdown_Step**	0,244	0,065	3,775	<0,001
**regiaonorte:Lockdown_Step**	0,045	0,064	0,697	0,486
**regiaosudeste:Lockdown_Step**	0,070	0,064	1,099	0,272

**regiaosul:Lockdown_Step**	-0,022	0,064	-0,348	0,728

2.7 Tabela suplementar dos resultados do teste de razão de verossimilhança entre os modelos com ou sem interação

Modelo	Df	AIC	BIC	logLik	deviance	Chisq	Chi Df	Pr(>Chisq)
**Sem interação**	12	7.164,35	7.210,99	-3.570,18	7.140,35	NA	NA	NA
**Com interação**	36	7.101,20	7.241,10	-3.514,60	7.029,20	111,158	24	<0,001

3. Detalhes da Raspagem de Dados

A raspagem de dados realizada no SISAB – seção “Produção Individual” – considerou exclusivamente os atendimentos classificados como “Atendimento Individual”. Para filtrar os atendimentos a transtornos mentais, foram adicionados os seguintes códigos na seção “CIAP/CID”:

F000	F001	F011	F012
F02	F020	F028	F03
F058	F059	F063	F064
F068	F069	F078	F079
F100	F106	F110	F116
F121	F126	F131	F136
F141	F146	F151	F156
F161	F166	F181	F186
F191	F196	F202	F203
F208	F209	F229	F23
F238	F239	F252	F258
F300	F301	F310	F311
F316	F317	F321	F322
F33	F330	F338	F339
F349	F38	F40	F400
F41	F410	F419	F42
F429	F43	F439	F44
F444	F445	F449	F45

F454	*F458*	*F488*	*F489*
F503	*F504*	*F51*	*F510*
F515	*F518*	*F522*	*F523*
F527	*F528*	*F538*	*F539*
F600	*F601*	*F606*	*F607*
F620	*F621*	*F631*	*F632*
F64	*F640*	*F65*	*F650*
F655	*F656*	*F661*	*F662*
F681	*F688*	*F708*	*F709*
F719	*F72*	*F73*	*F730*
F780	*F781*	*F791*	*F798*
F802	*F803*	*F811*	*F812*
F82	*F83*	*F843*	*F844*
F88	*F89*	*F909*	*F91*
F918	*F919*	*F93*	*F930*
F939	*F94*	*F949*	*F95*
F959	*F98*	*F984*	*F985*
F99	*F00*	*F010*	*F019*
F024	*F051*	*F062*	*F067*
F072	*F10*	*F105*	*F11*
F115	*F120*	*F125*	*F130*
F135	*F140*	*F145*	*F150*
F155	*F160*	*F165*	*F180*

F185	*F190*	*F195*	*F201*
F206	*F228*	*F233*	*F251*
F30	*F31*	*F315*	*F320*
F329	*F334*	*F348*	*F39*
F409	*F418*	*F428*	*F438*
F443	*F448*	*F453*	*F481*
F502	*F509*	*F514*	*F521*
F526	*F531*	*F60*	*F605*
F62	*F630*	*F639*	*F649*
F654	*F660*	*F680*	*F701*
F718	*F729*	*F78*	*F790*
F801	*F810*	*F819*	*F842*
F849	*F908*	*F913*	*F929*
F938	*F948*	*F958*	*F983*
F989	*F111*	*F117*	*F122*
F127	*F132*	*F137*	*F142*
F147	*F152*	*F157*	*F162*
F167	*F182*	*F187*	*F192*
F197	*F101*	*F107*	*F002*
F013	*F021*	*F04*	*F06*
F065	*F07*	*F09*	*F204*
F21	*F230*	*F24*	*F259*
F302	*F312*	*F318*	*F323*

F331	*F34*	*F380*	*F401*
F411	*F420*	*F430*	*F440*
F446	*F450*	*F459*	*F50*
F505	*F511*	*F519*	*F524*
F529	*F54*	*F602*	*F608*
F628	*F633*	*F641*	*F651*
F658	*F668*	*F69*	*F71*
F720	*F731*	*F788*	*F799*
F808	*F813*	*F84*	*F845*
F90	*F910*	*F92*	*F931*
F940	*F950*	*F980*	*F986*
F112	*F118*	*F123*	*F128*
F133	*F138*	*F143*	*F148*
F153	*F158*	*F163*	*F168*
F183	*F188*	*F193*	*F198*
F102	*F108*	*F009*	*F018*
F022	*F05*	*F060*	*F066*
F070	*F20*	*F205*	*F22*
F231	*F25*	*F28*	*F308*
F313	*F319*	*F328*	*F332*
F340	*F381*	*F402*	*F412*
F421	*F431*	*F441*	*F447*
F451	*F48*	*F500*	*F508*

F512	*F52*	*F525*	*F53*
F55	*F603*	*F609*	*F629*
F638	*F642*	*F652*	*F659*
F669	*F70*	*F710*	*F721*
F738	*F789*	*F80*	*F809*
F818	*F840*	*F848*	*F900*
F911	*F920*	*F932*	*F941*
F951	*F981*	*F988*	*F113*
F119	*F124*	*F129*	*F134*
F139	*F144*	*F149*	*F154*
F159	*F164*	*F169*	*F184*
F189	*F194*	*F199*	*F103*
F109	*F01*	*F023*	*F050*
F061	*F071*	*F200*	*F220*
F232	*F250*	*F29*	*F309*
F314	*F32*	*F333*	*F341*
F388	*F408*	*F413*	*F422*
F432	*F442*	*F452*	*F480*
F501	*F513*	*F520*	*F530*
F59	*F604*	*F61*	*F63*
F648	*F653*	*F66*	*F68*
F700	*F711*	*F728*	*F739*
F79	*F800*	*F81*	*F841*

F901	*F912*	*F928*	*F933*
F942	*F952*	*F982*	*F114*
F12	*F13*	*F14*	*F15*
F16	*F18*	*F19*	*F104*
