# Supplementary material for: Trends in mental health care in Brazilian Primary Health Care before, during, and after COVID-19: an ecological interrupted time-series study (2018-2023)
Source: Rev Bras Epidemiol. 2026 Jul 31;29:e260037. doi: 10.1590/1980-549720260037 (PMC13427217; doi:10.1590/1980-549720260037)
Supplement: Translation

# Translation: Tendências no cuidado aos transtornos mentais na atenção primária no Brasil antes, durante e após a covid-19: estudo ecológico de séries temporais interrompidas (2018-2023)

**DOI:** 10.1590/1980-549720260037.2

**Authors:** Lucas Oltramari, Milenne Souza de Lima, Eduardo Viegas da Silva

**Affiliations:** I Escola de Saúde Pública do Rio Grande do Sul - Porto Alegre (RS), Brasil.; II Secretaria Municipal de Saúde de Cachoeirinha - Cachoeirinha (RS), Brasil.; III Secretaria Estadual de Saúde do Rio Grande do Sul - Porto Alegre (RS), Brasil.

**Keywords:** Transtornos mentais, Atenção primária à saúde, Pesquisa de serviços de saúde, Covid-19, Análise de séries temporais interrompidas

## Abstract

**Objetivo:**

Avaliar a evolução temporal dos atendimentos por transtornos mentais na Atenção Primária à Saúde (APS) do Sistema Único de Saúde (SUS) no Brasil e estimar os efeitos do distanciamento social, da pandemia e do período pós-pandêmico de covid-19.

**Métodos:**

Estudo ecológico nacional de séries temporais interrompidas utilizando dados do Sistema de Informação em Saúde para a Atenção Básica, do Departamento de Informação e Informática do SUS (Sisab/DataSUS). Analisaram-se as taxas mensais proporcional e de incidência populacional de atendimentos por transtornos mentais (CID-10 F00-F99) na APS. Ajustaram-se modelos de regressão segmentada com distribuição binomial negativa e estrutura autorregressiva.

**Resultados:**

Foram analisados 38,7 milhões de atendimentos. No período pré-pandemia, observou-se tendência crescente da participação dos transtornos mentais nos atendimentos da APS (exp(β)_Tendência_=1,011; IC95% 1,008-1,014). Durante o distanciamento social, ocorreu aumento abrupto da proporção desses atendimentos (exp(β)_Nível_=1,241; IC95% 1,149-1,339), em grande parte associado à redução do volume total de atendimentos na APS. No restante da pandemia, a elevação proporcional estagnou. Houve heterogeneidade regional (p<0,001), com maior elevação de nível no Nordeste. Já no período pós-pandêmico, observou-se retomada do crescimento tanto da proporção dos atendimentos quanto da incidência populacional de atendimentos por transtornos mentais; entre os grupos diagnósticos, destacou-se o crescimento dos transtornos ansiosos, do desenvolvimento e infantojuvenis.

**Conclusão:**

A participação dos transtornos mentais nos atendimentos da APS brasileira aumentou ao longo do período analisado. O distanciamento social produziu elevação proporcional abrupta em contexto de retração de outros atendimentos, seguida de recomposição heterogênea entre regiões e grupos diagnósticos. Os achados indicam aumento recente da demanda por cuidado em saúde mental na APS.

## INTRODUÇÃO

Entre os usuários da Atenção Primária à Saúde (APS), a prevalência de transtornos mentais, especialmente Transtornos Mentais Comuns (TMC), como a ansiedade e a depressão, é elevada^1^ ^,^ ^2^ ^,^ ^3^. Os transtornos mentais figuram também entre os principais responsáveis pelo ônus global de incapacidade, com destaque para o Brasil, que lidera indicadores de anos de vida perdidos por incapacidade por transtornos ansiosos e humor bipolar nas Américas^4^ ^,^ ^5^ ^,^ ^6^.

No contexto do Sistema Único de Saúde (SUS), o cuidado em saúde mental é organizado pela Rede de Atenção Psicossocial (Raps), que integra múltiplos pontos de atenção. Enquanto os Centros de Atenção Psicossocial (Caps) manejam casos graves e persistentes, a APS é responsável pelo cuidado de TMC e pelo acompanhamento longitudinal de casos estabilizados, desempenhando papel estratégico na detecção precoce e coordenação do cuidado; em linha com as recomendações da Organização Mundial da Saúde (OMS) de fomentar serviços comunitários, especialmente em países de média renda^7^ ^,^ ^8^.

Dado o papel central da APS na coordenação do cuidado aos transtornos mentais no âmbito da Raps, torna-se relevante compreender como esses agravos se expressam na produção assistencial desse nível de atenção. Embora haja diversos estudos qualitativos sobre o cuidado à saúde mental na APS^9^, a literatura nacional ainda é escassa quanto à frequência e à participação proporcional desses atendimentos ao longo do tempo.

Adicionalmente, a pandemia de covid-19 representou uma ruptura nos sistemas de saúde, incluindo períodos de distanciamento social e reorganização da oferta, com impactos relevantes sobre a saúde mental da população^10^ e sobre o uso dos serviços^11^ ^,^ ^12^. Estudos internacionais indicam elevações pontuais da demanda proporcional de atendimento a transtornos mentais na APS durante os primeiros meses da pandemia^13^. No Brasil, há evidências de aumento das desigualdades em sintomas de saúde mental durante a pandemia. O período de distanciamento social ocasionou perdas financeiras, em especial entre famílias de mais baixa posição socioeconômica. Esta e outras exposições, relacionadas ao espalhamento do vírus SARS-CoV-2 e às medidas implementadas para conter aumentos abruptos na incidência de covid-19, possivelmente influenciaram prevalências de TMC no nível populacional, o que potencialmente elevou a necessidade de atendimentos na APS^14^. No entanto, permanece incerto como esse contexto se refletiu na organização e no perfil dos atendimentos realizados na APS do SUS, particularmente no que se refere à participação relativa dos transtornos mentais no conjunto da assistência prestada.

Assim, o objetivo do estudo foi avaliar a evolução temporal da taxa de participação proporcional e da taxa de incidência populacional de atendimentos a transtornos mentais na APS, para o Brasil e suas macrorregiões, estimando efeitos do período mais crítico de distanciamento social, da pandemia como um todo e do pós-pandemia de covid-19. Adicionalmente, a estratificação por grupos de transtornos mentais buscou identificar possíveis diferenças entre seus padrões temporais específicos.

## MÉTODOS

### Desenho do estudo e fontes de dados

Trata-se de um estudo ecológico de séries temporais interrompidas^15^, com dados populacionais de atendimentos por transtornos mentais na APS do SUS para cada mês, entre janeiro de 2018 e dezembro de 2023. O estudo tem abrangência nacional, cobrindo o Brasil e suas cinco macrorregiões. As informações foram extraídas da seção “Produção Individual” do Sistema de Informações em Saúde para a Atenção Básica (Sisab) do Departamento de Informática do SUS (DataSUS), que consolida registros de atendimentos realizados diretamente por profissionais da atenção primária no e-SUS APS.

### Instrumentos de coleta de dados

Para a coleta dos dados, foi empregada uma estratégia de raspagem automatizada programada em *Python* (versão 3.12), a fim de garantir abrangência e reprodutibilidade no acesso às informações disponibilizadas. A extração foi realizada em maio de 2025.

### Variáveis e definições

Para todas as análises, os dados foram estratificados em:

1. Unidade geográfica: atendimentos por transtornos mentais no Brasil e em cada macrorregião; e
2. Atendimentos de transtornos mentais e comportamentais, agregados a nível de grupo segundo a CID-10, em nível nacional.

O estudo foi segmentado em quatro fases:

1. Pré-pandemia (jan./2018-mar./2020);
2. Distanciamento social (abr.-jun./2020);
3. Pandemia (jul./2020-mar./2022); e
4. Pós-pandemia (abr./2022-dez./2023).

Os períodos foram definidos com base na identificação de ruptura estrutural sustentada a partir de abril de 2020 e nos principais marcos normativos nacionais (Decreto Legislativo n^o^ 6/2020; Portaria GM/MS n^o^ 356/2020; Portaria GM/MS n^o^ 913/2022). O distanciamento social foi analisado separadamente devido à queda abrupta e transitória no volume total de atendimentos, potencialmente capaz de distorcer as estimativas da fase pandêmica subsequente.

A variável desfecho foi a taxa proporcional mensal de registros com diagnóstico principal de transtornos mentais (CID-10 F00-F99), calculada como o número de atendimentos por transtornos mentais dividido pelo total de atendimentos gerais e multiplicado por mil. O indicador permite avaliar a participação relativa desses transtornos no conjunto da assistência prestada na APS ao longo do tempo. As variáveis independentes incluíram o tempo contínuo em meses desde janeiro de 2018, além de indicadores que capturaram alterações de nível e tendência associadas às segmentações por fase do período.

### Análise estatística

#### Análise descritiva

A análise descritiva consistiu na apresentação de medidas absolutas e relativas de atendimentos por transtornos mentais na APS. Foram dispostas medianas e Intervalos Interquartis (IQR) para os segmentos temporais. As proporções e taxas proporcionais de atendimento foram calculadas em relação ao total de atendimentos na APS na respectiva unidade geográfica.

#### Modelo de regressão segmentada para taxas proporcionais de atendimento

Para quantificar o impacto das fases de distanciamento social, da pandemia e pós-pandêmica sobre as taxas proporcionais de atendimentos por transtornos mentais na APS, foram ajustados modelos lineares generalizados mistos com estimação de quase-verossimilhança, utilizando a função *glmmPQL* do pacote *nlme*, especificados com distribuição binomial negativa e ligação logarítmica, considerando a sobredispersão observada. O parâmetro de dispersão foi estimado de forma iterativa com base no método dos momentos. O modelo incluiu estrutura de correlação ARMA(1,1) nos resíduos ao longo do tempo. Para controlar padrões sazonais, foram incluídos termos harmônicos de *Fourier* no componente fixo do modelo. Cada modelo utilizou como variável dependente o número mensal de atendimentos por transtornos mentais, incluindo como *offset* o logaritmo do total de atendimentos gerais no mesmo período.

Para avaliar formalmente diferenças regionais na resposta aos períodos segmentados, ajustou-se modelo complementar via *glmmTMB*, com distribuição binomial negativa e estrutura de correlação AR(1) nos resíduos, incluindo a variável região como efeito fixo e termos de interação entre região e os indicadores de fase temporal. A comparação entre modelos com e sem interação foi realizada por teste de razão de verossimilhança (nível de significância de 5%). Todas as análises foram realizadas no *R* (v.4.5.0).

Os coeficientes de mudança de nível foram interpretados como alterações estruturais na proporção, enquanto os de mudança de tendência refletiram modificações graduais, ambos comparados ao período pré-pandemia. Para o período de distanciamento social, modelou-se apenas uma mudança imediata de nível, sem estimativa de tendência, em razão de sua curta duração. Para os períodos pandêmico e pós-pandêmico, foram incluídos termos de mudança de nível e de tendência. Adotou-se nível de significância de 5% (p<0,05). Detalhes adicionais sobre as modelagens estão descritos no material suplementar.

#### Modelo de regressão segmentada para taxas populacionais de atendimento

Adicionalmente, estimaram-se modelos utilizando o número mensal de atendimentos por transtornos mentais como variável dependente, incluindo como *offset* o logaritmo da população residente estimada para cada região, segundo dados do Censo Demográfico 2022 do Instituto Brasileiro de Geografia e Estatística (IBGE). Foram também ajustados modelos tendo como variável dependente o número mensal total de atendimentos na APS. Esses modelos mantiveram a estrutura de regressão segmentada adotada na análise por taxas proporcionais, com o objetivo de avaliar se os achados poderiam ser influenciados por variações no volume total de atendimentos da APS ao longo do período.

### Aspectos éticos

Sendo os dados utilizados de natureza agregada, de domínio público e sem identificação de indivíduos, o estudo está dispensado de aprovação por Comitê de Ética em Pesquisa, conforme a Resolução n^o^ 510/2016, do Conselho Nacional de Saúde.

## Declaração de disponibilidade de dados:

Os dados que dão suporte aos resultados derivam de fontes públicas (Sisab/DataSUS). O conjunto de dados integrado e processado pelos autores está disponível mediante solicitação ao autor correspondente.

## RESULTADOS

### Análise descritiva

Entre janeiro de 2018 e dezembro de 2023, foram registrados 38,7 milhões de atendimentos com diagnóstico principal de transtorno mental na APS. A mediana das proporções mensais destes atendimentos, nacionalmente, aumentou de 1,90% no período pré-pandêmico para 2,88% do total de atendimentos na APS no pós-pandemia (Tabela 1).

**Tabela 1.** Número e proporção de atendimentos a transtornos mentais na Atenção Primária a Saúde (Brasil e macrorregiões, 2018-2023; mediana mensal intervalo interquartil).

Unidade geográfica	Total (2018 a 2023)		Pré-pandemia		Distanciamento social		Pandemia		Pós-pandemia	
	Atendimentos	% APS*	Mediana mensal (IQR)	% APS*	Mediana mensal (IQR)	% APS*	Mediana mensal (IQR)	% APS*	Mediana mensal (IQR)	% APS*
Brasil	38795399	2,46	360686,5 (78590,25)	1,90	344455,0 (48529,0)	2,78	509752,0 (93478,0)	2,51	789196,0 (204127,0)	2,88
Centro-Oeste	3029967	2,61	29369,5 (6284,5)	2,25	31097,0 (3801,0)	2,98	38171,0 (7461,0)	2,58	62179,0 (13875,0)	2,97
Nordeste	7761815	1,99	62092,0 (14715,5)	1,15	65036,0 (5337,5)	2,09	100095,0 (26585,0)	2,06	174538,0 (42671,0)	2,68
Norte	1470280	1,44	13632,0 (2939,75)	1,08	13058,0 (1613,0)	1,52	19523,0 (5453,0)	1,32	30268,0 (7962,0)	1,79
Sudeste	17269462	2,71	161956,5 (42904,25)	2,10	148107,0 (25140,0)	3,08	233185,0 (33709,0)	2,83	353048,0 (86025,0)	3,05
Sul	9263875	2,79	95571,0 (15018,5)	2,65	87157,0 (12637,5)	3,37	118612,0 (26835,0)	2,76	172807,0 (40856,0)	2,96

*proporção entre atendimentos a transtornos mentais e o total de atendimentos na Atenção Primária a Saúde.

APS: Atenção Primária a Saúde; IQR: intervalo interquartil.

Fonte: Elaboração própria.

Regionalmente, Sul e Sudeste apresentaram as maiores proporções ao longo do período, enquanto Norte e Nordeste partiram de níveis mais baixos, com crescimento mais acentuado neste último.

#### Análise dos modelos de regressão segmentada para taxas proporcionais de atendimento

A Tabela 2 demonstra as estimativas da tendência mensal no período pré-pandemia e os efeitos de mudança de nível e de tendência associados às fases subsequentes. Nos períodos de distanciamento social, pandemia e pós-pandemia, os coeficientes exponenciados (exp(β)) representam razões multiplicativas na proporção de atendimentos por transtornos mentais, tendo o período pré-pandemia como referência.

**Tabela 2.** Estimativas de mudança de nível e tendência na proporção de atendimentos por transtornos mentais na Atenção Primária a Saúde, associadas às fases da pandemia.

Estratificação	Efeito de mudança	Pré-pandemia		Distanciamento social		Pandemia		Pós-pandemia	
		exp(β)	IC95%	exp(β)	IC95%	exp(β)	IC95%	exp(β)	IC95%
Unidades geográficas									
Brasil	Nível			1,241	1,149-1,339	1,079	1,005-1,158	0,855	0,760-0,961
	Tendência	1,011	1,008-1,014			0,989	0,984-0,994	1,001	0,996-1,006
Centro-Oeste	Nível			1,179	1,065-1,306	0,977	0,885-1,079	0,877	0,744-1,033
	Tendência	1,008	1,004-1,012			0,994	0,987-1,001	0,999	0,992-1,006
Nordeste	Nível			1,404	1,271-1,551	1,263	1,142-1,398	1,032	0,871-1,223
	Tendência	1,016	1,012-1,020			0,989	0,982-0,996	1,001	0,994-1,008
Norte	Nível			1,186	1,079-1,303	0,918	0,835-1,009	0,980	0,839-1,145
	Tendência	1,011	1,007-1,015			1,003	0,997-1,010	1,004	0,998-1,011
Sudeste	Nível			1,198	1,104-1,300	1,059	0,980-1,144	0,779	0,685-0,885
	Tendência	1,013	1,010-1,016			0,987	0,981-0,992	0,996	0,991-1,002
Sul	Nível			1,189	1,089-1,298	1,053	0,967-1,146	0,809	0,703-0,930
	Tendência	1,005	1,002-1,008			0,988	0,982-0,993	1,007	1,001-1,013
Grupos de transtornos (Brasil)									
Transtornos Mentais Orgânicos (F00-F09)	Nível			1,225	1,127-1,332	1,132	1,046-1,225	0,896	0,787-1,021
	Tendência	1,008	1,005-1,011			0,987	0,982-0,993	0,997	0,992-1,003
Transtornos Mentais Devido ao Uso de Substância Psicoativa (F10-F19)	Nível			1,246	1,148-1,352	1,181	1,096-1,272	0,898	0,795-1,015
	Tendência	1,009	1,006-1,011			0,984	0,979-0,989	1,007	1,002-1,012
Esquizofrenia e Transtornos Delirantes (F20-F29)	Nível			1,214	1,114-1,323	1,041	0,954-1,136	0,785	0,678-0,908
	Tendência	1,007	1,003-1,010			0,987	0,981-0,993	1,001	0,995-1,007
Transtornos do Humor (F30-F39)	Nível			1,279	1,183-1,384	1,057	0,985-1,135	0,736	0,655-0,828
	Tendência	1,008	1,005-1,011			0,983	0,978-0,988	0,994	0,989-0,999
Transtornos de Ansiedade (F40-F48)	Nível			1,366	1,254-1,488	1,195	1,101-1,298	0,946	0,825-1,085
	Tendência	1,015	1,011-1,018			0,989	0,983-0,995	1,000	0,994-1,005
Transtornos Fisiológicos (F50-F59)	Nível			1,425	1,323-1,535	1,279	1,194-1,370	0,685	0,611-0,767
	Tendência	1,011	1,008-1,014			0,971	0,966-0,976	0,992	0,987-0,996
Transtornos de Personalidade (F60-F69)	Nível			1,003	0,912-1,103	1,023	0,938-1,117	0,635	0,548-0,734
	Tendência	1,021	1,018-1,025			0,982	0,976-0,988	0,991	0,986-0,997
Deficiência Intelectual (F70-F79)	Nível			0,794	0,709-0,889	0,817	0,738-0,906	0,862	0,728-1,022
	Tendência	1,007	1,003-1,011			1,006	0,999-1,014	1,006	0,998-1,013
Transtornos do Desenvolvimento (F80-F89)	Nível			0,395	0,336-0,465	0,565	0,483-0,661	0,863	0,666-1,119
	Tendência	1,015	1,009-1,021			1,025	1,014-1,036	1,011	1,000-1,022
Transtornos do Comportamento e Emocionais da Infância ou Adolescência (F90-F98)	Nível			0,845	0,749-0,953	0,815	0,725-0,916	1,005	0,828-1,220
	Tendência	1,002	0,997-1,007			1,010	1,002-1,019	1,021	1,013-1,029
Transtorno Mental Não Especificado (F99)	Nível			1,569	1,403-1,754	1,133	0,999-1,284	0,507	0,409-0,629
	Tendência	1,026	1,021-1,031			0,964	0,956-0,973	0,985	0,976-0,994

exp(β): razão de taxas estimada pelo modelo; IC95%: intervalo de confiança de 95%.

Valores em negrito indicam significância estatística (p<0,05).

Fonte: Elaboração própria.

No Brasil, a proporção de atendimentos por transtornos mentais apresentava pequeno crescimento mensal significativo ao longo do período pré-pandemia (exp(β)_Tendência_=1,011; Intervalo de Confiança 95% [IC95%] 1,008-1,014). Durante o período de distanciamento social, observou-se aumento imediato no nível da série (exp(β)_Nível_=1,241; IC95% 1,149-1,339). Durante o restante da pandemia, o nível manteve-se acima do contrafactual (exp(β)_Nível_=1,079; IC95% 1,005-1,158), com redução da tendência mensal (exp(β)_Tendência_=0,989; IC95% 0,984-0,994). No pós-pandemia, observou-se nível inferior ao contrafactual (exp(β)_Nível_=0,855; IC95% 0,760-0,961), sem alteração significativa da inclinação mensal.

As regiões seguiram padrão geral semelhante ao nacional, com aumento significativo de nível no período de distanciamento social: o Nordeste apresentou o maior incremento (exp(β)_Nível_=1,404; IC95% 1,271-1,551), enquanto o Centro-Oeste registrou o menor (exp(β)_Nível_=1,179), com valores intermediários nas demais regiões. Durante a pandemia, apenas o Nordeste manteve aumento significativo de nível (exp(β)_Nível_=1,263; IC95% 1,142-1,398), ao passo que Sudeste e Sul apresentaram desaceleração da tendência mensal. No período pós-pandêmico, Sudeste e Sul exibiram quedas de nível, enquanto Norte e Nordeste mantiveram trajetória ascendente. A heterogeneidade regional foi relevante, com forte evidência estatística para o termo de interação (p<0,001).

Transtornos ansiosos e do humor exibiram aumento abrupto de nível no período de distanciamento social, mais pronunciado para transtornos ansiosos (exp(β)_Nível_=1,366; IC95% 1,254-1,488) do que para transtornos do humor (exp(β)_Nível_=1,279; IC95% 1,183-1,384). Durante a pandemia, mantiveram aumento significativo de nível os transtornos ansiosos (exp(β)_Nível_=1,195; IC95% 1,101-1,298), além dos fisiológicos, relacionados ao uso de substâncias e orgânicos. Os transtornos do humor não diferiram do contrafactual. No pós-pandemia, os transtornos ansiosos estabilizaram-se em relação ao contrafactual, enquanto os transtornos do humor permaneceram abaixo do nível esperado. Transtornos do desenvolvimento e Transtornos do Comportamento e Emocionais com Início Habitual na Infância ou Adolescência (TCEIA) apresentaram queda inicial durante o distanciamento social e a pandemia, com recuperação progressiva. No pós-pandemia, os TCEIA destacaram-se por aceleração significativa da tendência (exp(β)_Tendência_=1,021; IC95% 1,013-1,029).

#### Análise dos modelos de regressão segmentada para taxas populacionais de atendimento

Observou-se tendência crescente significativa nas taxas de atendimentos por transtornos mentais no Brasil antes da pandemia (exp(β)_Tendência_=1,011; IC95% 1,010-1,013), enquanto as taxas totais da APS permaneceram estáveis, com padrão semelhante nas macrorregiões (Tabela 3). No período de distanciamento social, houve redução significativa das taxas totais da APS no Brasil (exp(β)_Nível_=0,609; IC95% 0,544-0,682), igualmente observada nas demais macrorregiões, ao passo que as taxas de atendimentos aos transtornos mentais apresentaram queda menos acentuada. Durante a pandemia, as taxas de saúde mental permaneceram estáveis, com recuperação gradual dos atendimentos totais. No pós-pandemia, verificou-se aumento das taxas de atendimentos aos transtornos mentais em algumas regiões, especialmente no Nordeste (exp(β)_Nível_=1,409; IC95% 1,284-1,545), concomitante à recomposição dos atendimentos totais (Figura 1).

**Tabela 3.** Estimativas de mudança de nível e tendência na taxa de incidência de atendimentos por transtornos mentais e de atendimentos totais na Atenção Primária a Saúde, associadas às fases da pandemia.

Estratificação	Efeito de mudança	Pré-pandemia		Distanciamento social		Pandemia		Pós-pandemia	
		exp(β)	IC95%	exp(β)	IC95%	exp(β)	IC95%	exp(β)	IC95%
Atendimentos totais na atenção primária									
Brasil	Nível			0,609	0,544-0,682	0,889	0,834-0,947	1,318	1,189-1,461
	Tendência	1,000	0,998-1,003			1,017	1,012-1,022	1,009	1,004-1,014
Centro-Oeste	Nível			0,675	0,604-0,753	0,833	0,783-0,887	1,158	1,047-1,280
	Tendência	1,007	1,005-1,009			1,014	1,010-1,019	1,002	0,998-1,007
Nordeste	Nível			0,581	0,522-0,646	0,831	0,787-0,877	1,239	1,132-1,356
	Tendência	1,000	0,997-1,002			1,017	1,013-1,021	1,008	1,004-1,012
Norte	Nível			0,623	0,555-0,699	0,929	0,874-0,987	1,190	1,076-1,316
	Tendência	1,001	0,999-1,004			1,014	1,009-1,018	1,007	1,002-1,012
Sudeste	Nível			0,644	0,545-0,761	0,908	0,786-1,050	1,354	1,065-1,721
	Tendência	1,000	0,994-1,006			1,017	1,007-1,028	1,011	1,001-1,021
Sul	Nível			0,593	0,534-0,658	0,885	0,834-0,938	1,337	1,216-1,469
	Tendência	1,002	1,000-1,004			1,019	1,015-1,024	1,008	1,004-1,012
Atendimentos a transtornos mentais (CID-10 F00-F99)									
Brasil	Nível			0,771	0,707-0,840	0,971	0,930-1,014	1,130	1,051-1,214
	Tendência	1,011	1,010-1,013			1,005	1,002-1,009	1,011	1,008-1,014
Centro-Oeste	Nível			0,824	0,766-0,887	0,832	0,804-0,862	1,034	0,974-1,097
	Tendência	1,014	1,013-1,016			1,008	1,006-1,011	1,003	1,000-1,006
Nordeste	Nível			0,964	0,866-1,073	1,085	1,026-1,147	1,409	1,284-1,545
	Tendência	1,013	1,011-1,016			1,009	1,004-1,013	1,011	1,007-1,015
Norte	Nível			0,788	0,710-0,875	0,834	0,791-0,880	1,186	1,086-1,296
	Tendência	1,011	1,009-1,013			1,021	1,017-1,025	1,013	1,009-1,017
Sudeste	Nível			0,763	0,646-0,901	0,952	0,822-1,102	1,036	0,813-1,320
	Tendência	1,014	1,008-1,019			1,004	0,994-1,014	1,007	0,997-1,018
Sul	Nível			0,763	0,661-0,882	0,918	0,809-1,041	1,097	0,890-1,351
	Tendência	1,006	1,001-1,011			1,010	1,001-1,019	1,016	1,007-1,025
Grupos de transtornos (Brasil)									
Transtornos Mentais Orgânicos (F00-F09)	Nível			0,778	0,715-0,846	1,026	0,984-1,069	1,208	1,126-1,295
	Tendência	1,008	1,007-1,010			1,004	1,000-1,007	1,007	1,004-1,010
Transtornos Mentais Devido ao Uso de Substância Psicoativa (F10-F19)	Nível			0,814	0,716-0,925	1,077	1,000-1,159	1,250	1,110-1,408
	Tendência	1,008	1,005-1,011			1,001	0,996-1,006	1,017	1,012-1,022
Esquizofrenia e Transtornos Delirantes (F20-F29)	Nível			0,796	0,704-0,900	0,927	0,857-1,002	1,079	0,954-1,220
	Tendência	1,006	1,003-1,009			1,006	1,001-1,011	1,011	1,006-1,017
Transtornos do Humor (F30-F39)	Nível			0,789	0,725-0,858	0,938	0,899-0,979	0,957	0,891-1,028
	Tendência	1,009	1,007-1,011			0,999	0,996-1,003	1,003	1,000-1,007
Transtornos de Ansiedade (F40-F48)	Nível			0,877	0,803-0,959	1,080	1,034-1,129	1,265	1,174-1,363
	Tendência	1,015	1,013-1,017			1,006	1,002-1,009	1,010	1,007-1,013
Transtornos Fisiológicos (F50-F59)	Nível			0,896	0,822-0,977	1,146	1,097-1,197	0,905	0,841-0,974
	Tendência	1,011	1,010-1,013			0,987	0,984-0,991	1,001	0,998-1,005
Transtornos de Personalidade (F60-F69)	Nível			0,637	0,538-0,754	0,904	0,779-1,048	0,824	0,644-1,054
	Tendência	1,022	1,016-1,028			1,000	0,990-1,011	1,001	0,991-1,011
Deficiência Intelectual (F70-F79)	Nível			0,466	0,408-0,531	0,745	0,692-0,802	1,125	0,999-1,268
	Tendência	1,007	1,004-1,010			1,023	1,018-1,029	1,017	1,012-1,023
Transtornos do Desenvolvimento (F80-F89)	Nível			0,241	0,192-0,303	0,499	0,404-0,615	1,088	0,769-1,539
	Tendência	1,016	1,007-1,024			1,043	1,028-1,059	1,021	1,007-1,036
Transtornos do Comportamento e Emocionais da Infância ou Adolescência (F90-F98)	Nível			0,521	0,433-0,627	0,713	0,605-0,839	1,276	0,974-1,672
	Tendência	1,003	0,997-1,010			1,029	1,017-1,040	1,030	1,019-1,042
Transtorno Mental Não Especificado (F99)	Nível			1,093	0,989-1,209	1,005	0,930-1,085	0,690	0,606-0,786
	Tendência	1,026	1,023-1,029			0,983	0,978-0,988	0,994	0,988-0,999

exp(β): razão de taxas estimada pelo modelo; IC95%: intervalo de confiança de 95%.

Valores em negrito indicam significância estatística (p<0,05).

Fonte: Elaboração própria.

**Figura 1.** Séries temporais de atendimentos por transtornos mentais na Atenção Primária a Saúde por unidade geográfica (2018-2023): (a) proporção de atendimentos; (b) incidência por população residente.

Fonte: Elaboração própria.

Na análise por grupos diagnósticos, observou-se aumento nos atendimentos por transtornos ansiosos, com elevação durante a pandemia e pós-pandemia, acompanhados de aumento da tendência temporal. No período pós-pandêmico, foram observados aumentos de tendência nos atendimentos por TCEIA e por transtornos do desenvolvimento psicológico (Figura 2). Resultados completos encontram-se no material suplementar.

**Figura 2.** Séries temporais de atendimentos na Atenção Primária a Saúde para grupos diagnósticos selecionados, Brasil (2018-2023): (a) proporção de atendimentos; (b) incidência por população residente.

Fonte: Elaboração própria.

## DISCUSSÃO

Este estudo identificou aumento progressivo da participação dos transtornos mentais nos atendimentos da Atenção Primária à Saúde no Brasil, tendência já presente antes da pandemia. O início da pandemia e o período de distanciamento social produziram mudança abrupta na composição dos atendimentos, com elevação proporcional dos registros de saúde mental. Observou-se heterogeneidade regional, com crescimento mais pronunciado no Nordeste. Entre os grupos diagnósticos, destacaram-se o aumento dos transtornos ansiosos e a aceleração recente de diagnósticos do desenvolvimento e da infância.

Durante o período de distanciamento social, houve queda expressiva no total de atendimentos da APS, reduzindo o denominador da razão analisada. Assim, o aumento proporcional dos atendimentos por transtornos mentais pode refletir não apenas elevação de demanda, mas também retração mais intensa de outros tipos de atendimento. A análise complementar por taxas de incidência sugere que os atendimentos em saúde mental sofreram menor redução relativa, indicando manutenção diferencial do cuidado, embora não se possa descartar a combinação entre aumento de demanda e restrição de oferta.

A segmentação temporal permitiu identificar que o choque inicial da pandemia e do distanciamento social produziu elevação abrupta na proporção de atendimentos, seguida de desaceleração da tendência. No pós-pandemia, observou-se retorno ao crescimento mensal prévio, ainda que com nível médio inferior ao contrafactual estimado. A análise por incidência sugere que esse resultado reflete, em parte, a recomposição mais rápida do volume total de atendimentos da APS em relação aos atendimentos por transtornos mentais após o choque inicial da pandemia. Destaca-se o novo padrão de aceleração observado nos TCEIA, indicando possível reconfiguração recente no perfil da demanda assistencial.

Regionalmente, observou-se crescimento mais pronunciado dos atendimentos por transtornos mentais no Nordeste ao longo do período analisado. Esse padrão pode refletir a centralidade estrutural da APS na organização do cuidado regional, marcada por ampla cobertura da Estratégia Saúde da Família e maior dependência da população em relação ao SUS, em contraste com o maior peso do setor privado no Sudeste e as restrições históricas do Norte - relacionadas a disponibilidade de profissionais e desafios geográficos^16^. Diferenças na organização regional da Raps também podem influenciar esse padrão. Em contextos em que serviços especializados, como os Caps, exercem maior protagonismo na porta de entrada ou continuidade do cuidado, parte da demanda por saúde mental pode se concentrar nesses dispositivos, reduzindo sua expressão relativa na APS. No pós-pandemia, o crescimento observado no Nordeste sugere possível reorganização do fluxo assistencial e maior protagonismo da APS no cuidado em saúde mental.

Quanto aos grupos diagnósticos, identificaram-se dois perfis distintos de resposta à pandemia. De um lado, alguns diagnósticos, que incluem tanto TMC quanto transtornos mentais graves, apresentaram aumento abrupto de proporção durante o período de distanciamento social, seguido de desaceleração da tendência, padrão consistente com evidências que apontam intensificação de sintomas ansiosos e depressivos em contextos de isolamento social^10^ ^,^ ^14^ ^,^ ^17^ ^,^ ^18^. Em contraste, transtornos diagnosticados predominantemente na infância ou dependentes de cuidadores para acesso aos serviços exibiram queda acentuada durante o período de distanciamento social, com recuperação gradual posterior, sugerindo barreiras transitórias de acesso durante a pandemia - fenômeno já documentado em outros países para, por exemplo, pacientes com Transtorno do Espectro Autista (TEA) e seus familiares^19^.

Entre esses grupos, os transtornos do humor e os transtornos ansiosos representam a maior parte dos atendimentos na APS e apresentaram aumento de proporção nos meses iniciais da pandemia, padrão também observado em outros países^13^. Embora os transtornos do humor e os ansiosos apresentassem níveis semelhantes no início da série, observou-se aumento desproporcional dos transtornos ansiosos ao longo dos anos, especialmente após 2020.

A análise por taxas populacionais fornece uma perspectiva adicional: enquanto alguns grupos diagnósticos apresentaram mudanças evidentes na incidência de atendimentos, essas alterações nem sempre se refletiram em mudanças equivalentes nas taxas proporcionais. Esse descompasso sugere que parte do aumento observado pode decorrer da ampliação do acesso ao cuidado. Entretanto, a comparação entre transtornos ansiosos e do humor mostra padrões distintos no modelo de incidência: embora ambos representem os grupos mais frequentes, os transtornos do humor acompanharam mais de perto a trajetória contrafactual estimada, enquanto os transtornos ansiosos permaneceram acima dessa trajetória contrafactual, sugerindo que a expansão observada na incidência dos atendimentos aos transtornos ansiosos dificilmente pode ser atribuída apenas à ampliação do acesso aos serviços. Cabe considerar, contudo, que os transtornos ansiosos e os transtornos de humor apresentam comorbidade elevada^20^; ademais, os transtornos depressivos do tipo ansioso são os mais comumente encontrados na APS^21^, possivelmente levando a sobreposições diagnósticas.

Outro aspecto relevante foi a ascendência dos atendimentos aos transtornos do desenvolvimento psicológico e aos TCEIA no pós-pandemia. Os transtornos do desenvolvimento, especialmente o TEA, tornaram-se mais frequentes ao longo do tempo em nível global^22^. Embora existam diferentes explicações para esse aumento, uma hipótese central é a ampliação dos critérios diagnósticos, com a noção de espectro e um limiar clínico mais inclusivo^23^; fenômeno que também pode explicar a maior prevalência de TDAH^24^. Fatores institucionais e normativos, como a vinculação de direitos sociais a diagnósticos formais, podem ter contribuído para a ampliação dos registros, embora essa possibilidade não possa ser testada diretamente neste estudo. Assim, é possível que parte do crescimento observado reflita mudanças nas práticas diagnósticas, não necessariamente alterações de demanda.

Ressalta-se a baixa proporção de atendimentos relacionados ao uso de substâncias psicoativas ao longo de todo o período. Embora se observe discreto aumento no pós-pandemia, a participação proporcional desses agravos na APS permanece reduzida. Esse padrão pode estar associado à centralização do cuidado em serviços especializados (como o Caps Álcool e Drogas), à estigmatização dos usuários e à persistência da lógica de internação como principal via terapêutica^25^ ^,^ ^26^. Além disso, a eventual baixa incorporação de estratégias sistemáticas de triagem e intervenções breves na APS pode contribuir para a subdetecção nos estágios iniciais, hipótese que merece investigação futura. Considerando a elevada prevalência e o impacto desses transtornos no Brasil^27^, os achados sugerem subaproveitamento da utilização da APS no manejo desses agravos.

Sugerimos investigações adicionais com foco no aspecto das práticas diagnósticas ou de registro, especialmente para corroborar os achados relacionados ao aumento de atendimentos aos transtornos de desenvolvimento, TCEIA e transtornos ansiosos. Além disso, investigações adicionais com foco geográfico específico nas regiões do Brasil que considerem tanto as políticas de saúde locais e seus impactos no cuidado à saúde mental, bem como as especificidades geográficas de demanda e acesso. Ainda, sugerem-se análises que realizem comparações com outros dispositivos da Raps, identificando se a APS vem absorvendo demanda reprimida desses elementos, em especial os Caps. Finalmente, investigações que avaliem o impacto da implementação do cuidado a transtornos mentais na APS sobre desfechos como taxas de internação ou óbitos são instrumentais para fundamentar decisões e priorizar investimentos na gestão da saúde mental pública.

Apesar da robustez do estudo, destaca-se como limitação a possível inflação da tendência pré-pandemia devido à ainda em curso implementação do Sisab (provável estabilização a partir de 2019)^28^, que pode ter elevado artificialmente a tendência do contrafactual, à medida que gradualmente foram incorporados ao sistema municípios com diferentes perfis e práticas de registro. Além disso, o modelo estatístico não incluiu variáveis como fatores demográficos, socioeconômicos ou políticas locais, o que limita atribuição de causalidade isolada ao evento da pandemia. Trata-se de estudo ecológico, portanto os resultados devem ser interpretados apenas a nível populacional.

Este estudo demonstra que os atendimentos por transtornos mentais vinham assumindo participação progressivamente maior na APS brasileira antes da pandemia, refletindo processo estrutural de integração da saúde mental nesse nível de atenção. O período inicial de distanciamento social foi marcado por alteração na composição dos atendimentos, decorrente sobretudo da retração mais intensa do volume total da APS, embora os atendimentos em saúde mental tenham apresentado redução relativamente menor. No período subsequente, observou-se retomada da trajetória prévia, de forma heterogênea entre macrorregiões, com destaque para a consolidação dos transtornos ansiosos como o mais frequente grupo diagnóstico. Em conjunto, os achados sugerem que a pandemia atuou como choque conjuntural em uma tendência estrutural já em curso, mais do que como elemento transformador da organização do cuidado em saúde mental na APS.

## Material Suplementar
